# Multi-gene-based investigation on the molecular phylogeny of the hypotrichous family Strongylidiidae (Protista, Ciliophora), with notes on the ontogeny of a new genus and new species

**DOI:** 10.1007/s42995-024-00243-z

**Published:** 2024-08-23

**Authors:** Wenya Song, Shijing Zhang, Yuqing Li, Honggang Ma, Qiyu Li, Xiaotian Luo, Khaled A. S. Al-Rasheid, Hunter N. Hines, Xiaoteng Lu

**Affiliations:** 1 Institute of Evolution & Marine Biodiversity, Ocean University of China, Qingdao, 266003 China; 2 Key Laboratory of Aquatic Biodiversity and Conservation of Chinese Academy of Sciences, Institute of Hydrobiology, Chinese Academy of Sciences, Wuhan, 430072 China; 3 Weishan Special Aquaculture Base, Jining, 277600 China; 4 Zoology Department, King Saud University, 11451 Riyadh, Saudi Arabia; 5 Harbor Branch Oceanographic Institute, Florida Atlantic University, Fort Pierce, Florida, 34946 USA; 6 Department of Biology, Shenzhen MSU-BIT University, Shenzhen, 518172 China

**Keywords:** Ciliates, Cytochrome c oxidase subunit I gene, Morphogenesis, Phylogeny, Ribosomal RNA gene, Taxonomy

## Abstract

Ciliates in the subclass Hypotrichia have long been difficult to classify as they are one of the most polymorphic and highly differentiated groups, leading to their systematics remaining unresolved. Phylogenetic relationships within the hypotrich family Strongylidiidae have been ambiguous due to discordance between the morphological and genetic data. In this study, a new strongylidiid genus *Heterouroleptus* is established, mainly based on the novel mode of origin of the ventral cirral rows: left ventral cirral row (LVR) originates from frontal–ventral–transverse cirral anlagen (FVTA) III (anterior portion), IV (middle portion), and V (rear portion); right ventral cirral row comes from the entire FVTA VI. A new species, *Heterouroleptus weishanensis* gen. nov., sp. nov., is investigated along with the morphometric and molecular data from a population of *Strongylidium wuhanense*. Eight new sequences and nuclear gene markers (single-gene and multi-gene) are provided to analyze the phylogenetic relationships of strongylidiids, with the COI gene utilized to uncover further genetic information at species level and below. The results reveal that: (1) Strongylidiidae is monophyletic and has a close relationship with Dorsomarginalia; (2) *Heterouroleptus* gen. nov. forms a clade that is sister to all the other strongylidiids; (3) *Hemiamphisiella* Foissner, 1988 and *Pseudouroleptus* Hemberger, 1985 should not be synonyms, and both genera should be subdivided due to their variable morphological characteristics; (4) LVR originating from three anlagen is a plesiomorphy of Strongylidiidae. The discovery of the origin of the LVR not only contributes to the establishment of the genus *Heterouroleptus*, but also helps to improve the diagnosis of the family Strongylidiidae.

**Supplementary Information:**

The online version contains supplementary material available at 10.1007/s42995-024-00243-z.

## Introduction

Ciliated protozoa are among the most complicated and morphologically diverse single-celled eukaryotes (Bharti and Kumar 2023; Corliss 1979; Ma et al. 2022a; Song et al. 2009; Wang et al. 2022a; Zhang et al. 2022a). Some are ideal biological tools and are used as model organisms in various fields of research (Dong et al. 2022; Foissner et al. 2002; Hu et al. 2019; Jin et al. 2023; Vannini et al. 2012). Hypotrichia Stein, 1859, the majority of which are dorsoventrally flattened and often surface-associated in benthic habitats from a variety of regions (Bharti and Kumar 2023; Chae et al. 2022; Foissner et al. 1999; Omar et al. 2022; Shao et al. 2023; Wang et al. 2023), are considered to be one of the most complex and highly differentiated ciliate groups due to various morphological features (Adl et al. 2012; Berger 2011; Luo et al. 2022; Song et al. 2023; Xu et al. 2022; Zhang et al. 2022c). In addition to the difficulties in determining morphological and morphogenetic differences between hypotrich species even after cell fixation and staining, molecular sequence data have thus far also proven ambiguous for accurately determining the evolutionary relationships of taxa within this group, in particular via the creation of phylogenetic trees using only a single marker such as the SSU rDNA gene.

As one of the families within Hypotrichia, Strongylidiidae Fauré-Fremiet, 1961 was erected over 50 years ago. In recent major revisions (Jankowski 2007; Lynn 2008; Lynn and Small 2002; Shi 1999a; Tuffrau and Fleury 1994), Strongylidiidae was treated as a junior synonym of Spirofilidae. Luo et al. (2018) reactivated the family Strongylidiidae for three genera, i.e., *Pseudouroleptus* Hemberger, 1985, *Strongylidium* Sterki, 1878 and *Hemiamphisiella* Foissner, 1988.

According to Luo et al. (2018), all *Strongylidium* species cluster together, whereas *Pseudouroleptus* and *Hemiamphisiella* are always intermingled with each other in the SSU rDNA tree, indicating the ambiguous systematics of these two genera. A single locus is not always sufficient to accurately resolve complex evolutionary relationships among ciliates, therefore, concatenated multi-gene datasets, e.g., SSU rDNA, ITS1-5.8S rDNA-ITS2 and LSU rDNA, can provide more insight (Chi et al. 2021; Gentekaki et al. 2014; Hewitt et al. 2003; Huang et al. 2016; Wang et al. 2021; Zhang et al. 2022b). However, with only a few sequences of strongylidiids available and a lack of morphological data and/or vouchered specimens for most of these, further exploration of the molecular phylogeny combined with morphological descriptions of members of these genera is needed.

The mitochondrial cytochrome c oxidase subunit I (COI) gene shows higher resolution than other genes in delimiting taxa at species level and below of some ciliate lineages (Park et al. 2019; Rataj and Vďačný 2021; Strüder-Kypke and Lynn 2010; Zhao et al. 2016). Since an effective primer set for the COI gene from hypotrichs (Spirotrichea) was designed only recently (Park et al. 2019), the availability of COI gene sequences in public databases (such as GenBank) are still relatively limited.

Here, we provide more insights into the phylogeny of the family Strongylidiidae based on multiple genes. The SSU rDNA, ITS1-5.8S rDNA-ITS2, LSU rDNA, and COI data of a new strongylidiid species, *H. weishanensis* gen. nov., sp. nov., and the ITS1-5.8S rDNA-ITS2, LSU rDNA and COI markers of the Weishan population of *S. wuhanense* Luo et al., 2018, are reported for the first time. We also provide details of the morphology, ontogenesis and molecular phylogeny of *H. weishanensis* gen. nov., sp. nov. With these new data, further light is shed on the systematic relationships of strongylidiids and hypotheses of evolutionary relationships within Strongylidiidae are posited.

## Materials and methods

### Sample collection, observation, and identification

**Fig. 1:**
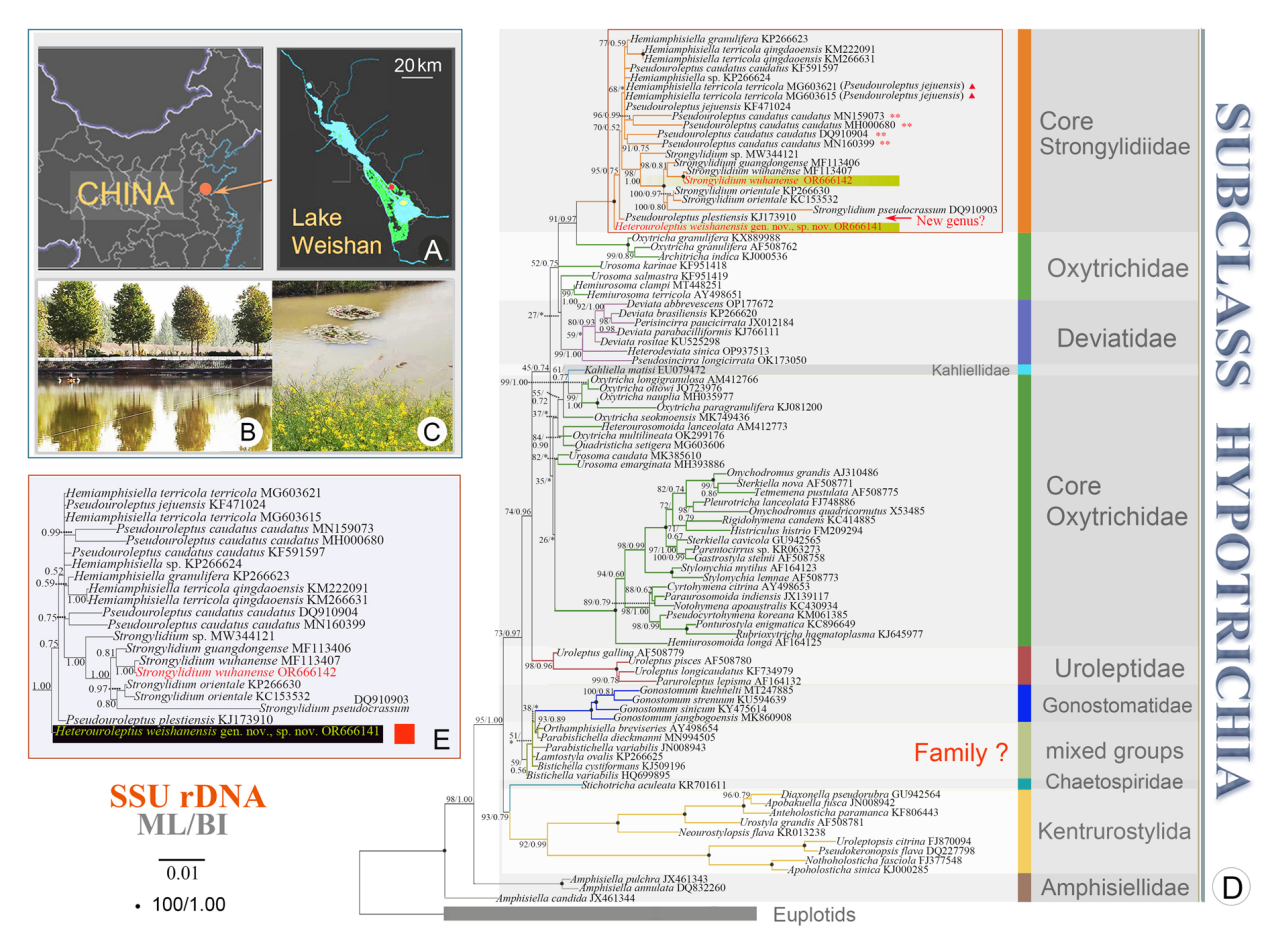
Sampling sites, maximum likelihood (ML), and BI phylogenetic tree inferred from SSU rDNA sequences. **A** Maps showing the location of sampling sites for the two species in Lake Weishan Wetland (red dot). **B**, **C** Photographs of the sampling sites for *Heterouroleptus weishanensis* gen. nov., sp. nov. (**B**) and *Strongylidium wuhanense* (**C**)*.* **D** ML phylogenetic tree. Newly sequenced species from this study are shown in red. Numbers near branches denote bootstrap values for ML and posterior probabilities for BI. Asterisks (*) indicate the disagreement between ML and BI trees. The scale bar corresponds to one substitution per 100 nucleotide positions. The “**” in red denotes that the validity of the sequence needs to be verified. The red triangles demonstrate two sequences of *Hemiamphisiella terricola terricola* (MG603621 and MG603615) should be conspecific with *Pseudouroleptus jejuensis*. The “?” indicates the family or genus assignment is questionable. **E** Branch of strongylidiids in BI tree

*Heterouroleptus weishanensis* gen. nov., sp. nov. was collected on June 29th, 2020, from a freshwater aquaculture pond in the Lake Weishan wetland (34°46′24.2″ N 117°09′42.1″ E), north-eastern China (Fig. 1). The salinity was < 0.5 and a water temperature was 15 ℃ (measured using a YSI, Xylem, model 10102030) at the time of sampling. Samples were collected following Kreutz and Foissner (2006). Populations of this species lasted only two days before collapsing, so clonal cultures could not be established. *S. wuhanense* was isolated on April 29th, 2019, from a small freshwater brook in the Lake Weishan wetland (34°46′24.2″ N, 117°09′42.1″ E). The salinity was < 0.5 and the water temperature was 16 ℃ (measured using a YSI, Xylem, model 10102030) at the time of sampling.

Cells were observed and recorded using an Olympus BX53 microscope and a DP74 camera following Foissner (2014). Protargol impregnation was according to Wilbert (1975). The protargol reagent was made in-house following Pan et al. (2013). Counts and measurements of prepared specimens were according to Song et al. (2023). Drawings of the morphogenetic processes followed Song et al. (2022). Classification was mainly according to Lynn (2008) and Paiva (2020). Terminology used was according to Berger (2008).

### DNA extraction, PCR amplification, and sequencing

Single cells of *H. weishanensis* gen. nov., sp. nov. and *S. wuhanense* were isolated and washed four times with distilled water to remove potential contamination. Genomic DNA was extracted from single cells using a DNeasy Blood & Tissue Kit (Qiagen, Hilden, Germany) following the manufacturer’s instruction. The SSU rDNA, ITS1-5.8S rDNA-ITS2, and LSU rDNA were amplified with the primers according to Lu et al. (2023). The PCR parameters were according to Lu et al. (2019). The COI gene was amplified, and the PCR parameters used, were according to Park et al. (2019). The resulting amplicons of the COI gene were purified using the EasyPure Quick Gel Extraction Kit (Transgen Biotech, China), then inserted into the pClone007 Blunt Simple Vector Kit (Tsingke Biological Technology, China), and transformed into DH5α Competent Cells (TransGen Biotech). Two clones for each species were arbitrarily selected and cultured in Luria–Bertani (LB) broth medium for 12 h, and then sequenced in both directions (Ma et al. 2022b). Q5 Hot Start High-Fidelity 2 × DNA Polymerase (New England BioLabs, MA, USA) was employed to minimize errors during PCR. Products of PCR were sequenced bidirectionally by the Tsingke Biological Technology Company (Qingdao, China). For each species, the fragments were assembled according to Song et al. (2023).

### Molecular phylogeny

**Fig. 2:**
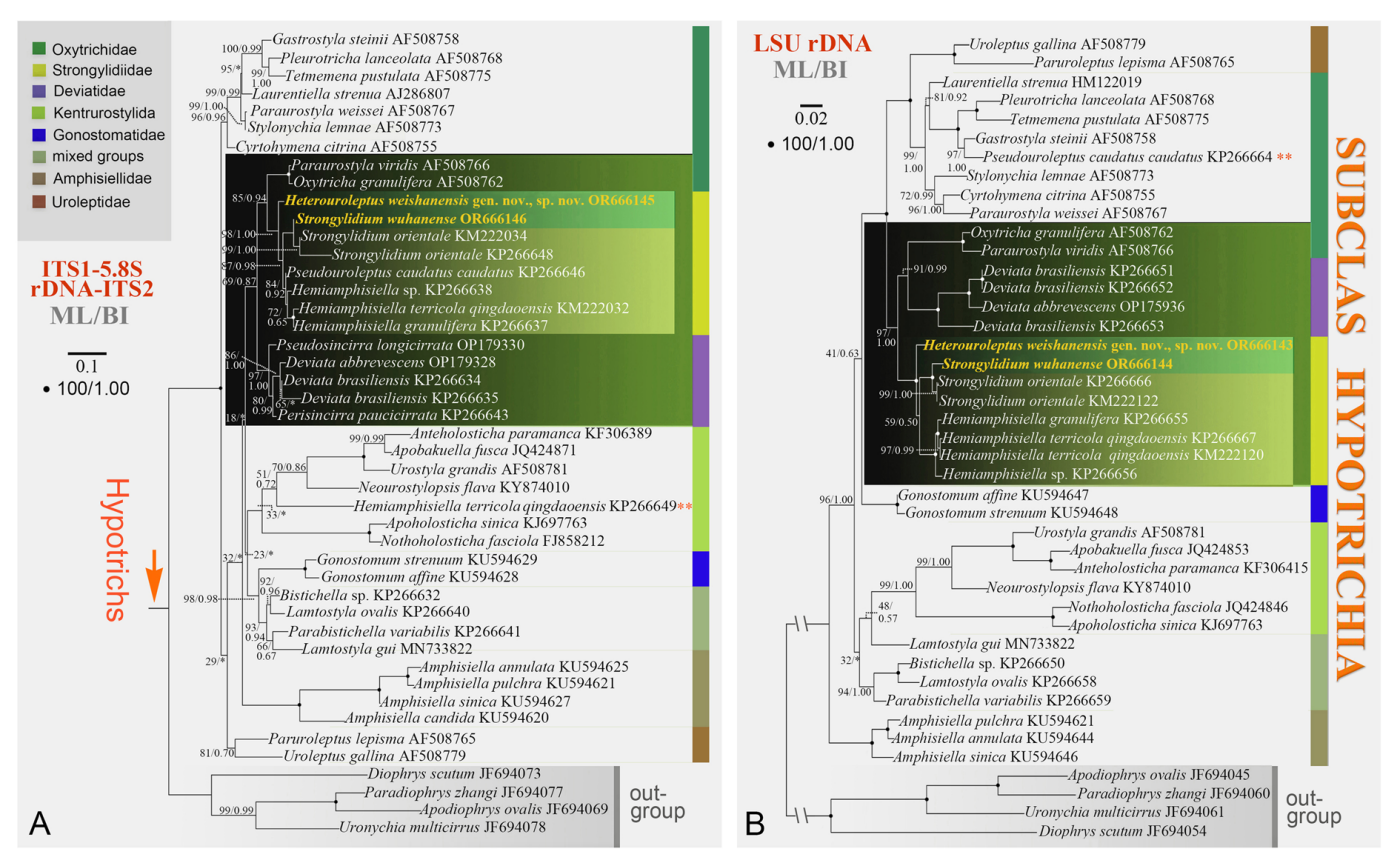
Maximum likelihood (ML) tree based on ITS1-5.8S rDNA-ITS2 (**A**) and LSU rDNA (**B**) sequence alignment. Newly sequenced species are shown in yellow. Numbers at the nodes represent the bootstrap values of ML out of 1000 replicates and the posterior probability values of Bayesian analysis (BI). Fully supported (100% ML/1.00 BI) branches are marked with solid circles. Asterisk (*) indicates disagreement between ML and BI analyses. The scale bars in **A** and **B** correspond to 10 and 2 substitutions per 100 nucleotide positions, respectively. The “**” in red denotes that the validity and/or the assignment of the sequence needs to be verified

**Fig. 3:**
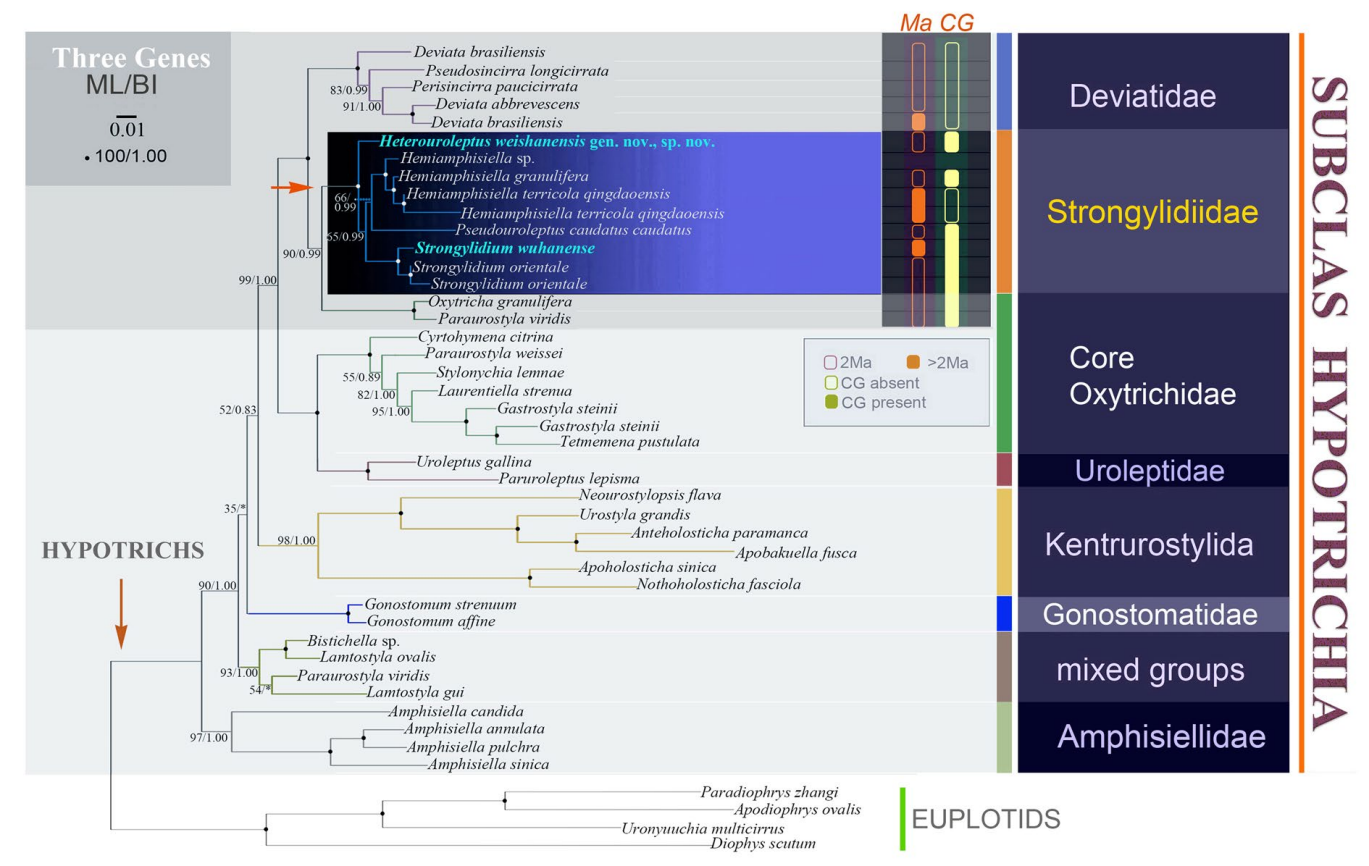
Phylogenetic tree based on concatenated sequences of SSU rDNA, ITS1-5.8S rDNA-ITS2 and LSU rDNA. Sequences from the present study are in blue. Numbers given at nodes of branches are Ultrafast bootstrap value (%) for ML analysis and posterior probabilities for BI analysis. Fully supported (100% ML/1.00 BI) branches are marked with solid circles. Asterisk (*) indicates disagreement between ML and BI analyses. The scale bar corresponds to one substitution per 100 nucleotide positions. *CG* cortical granules, *Ma* macronuclear nodules

**Fig. 4:**
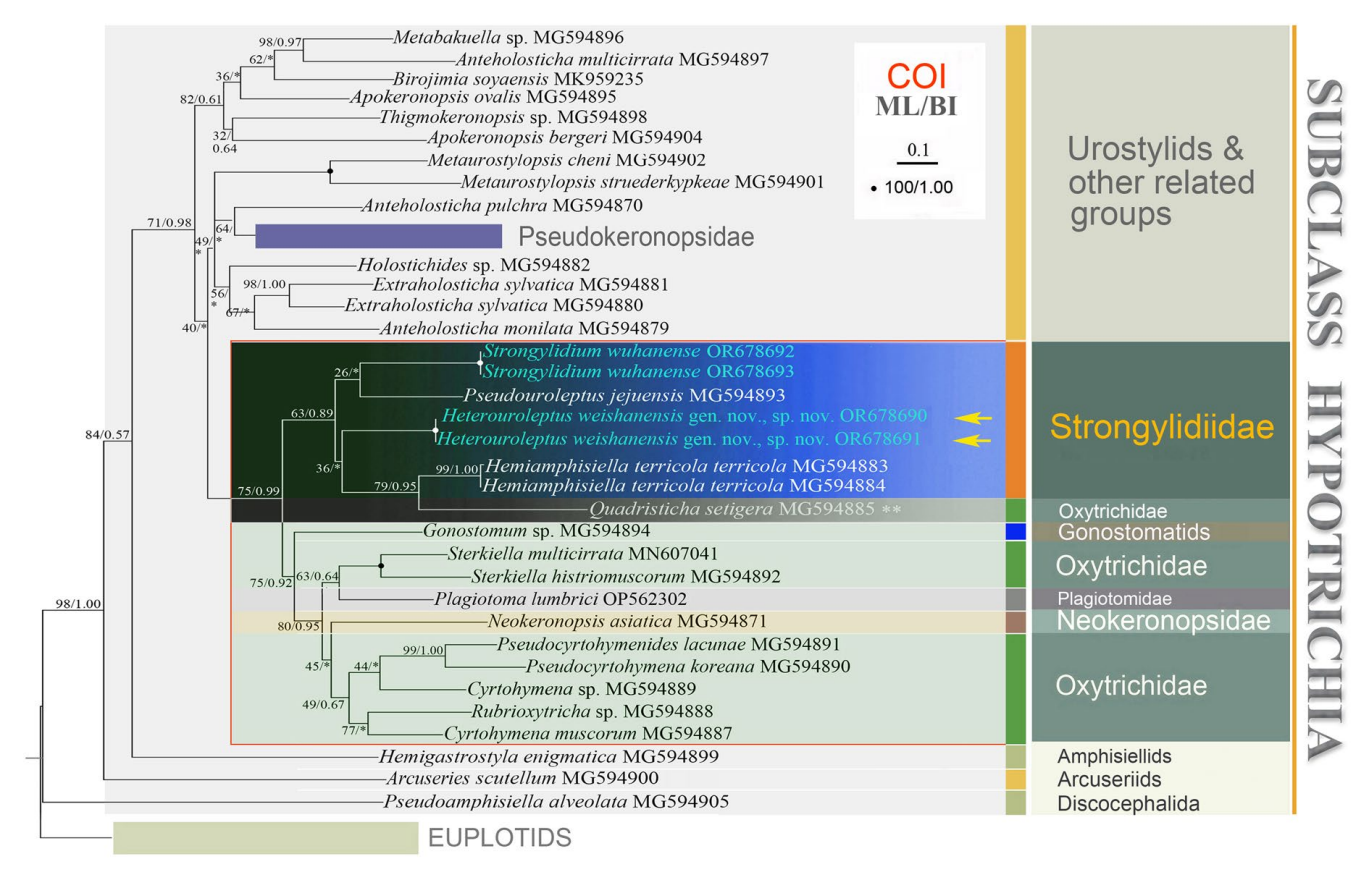
Phylogenetic tree based on mitochondrial cytochrome c oxidase subunit I (COI). Newly sequenced species in this work are shown in blue. Numbers at the nodes represent the bootstrap values of ML out of 1000 replicates and the posterior probability values of Bayesian analysis (BI). Fully supported (100% ML/1.00 BI) branches are marked with solid circles. Asterisk (*) indicates disagreement between ML and BI analyses. The scale bar corresponds to ten substitutions per 100 nucleotide positions. The “**” denotes that the validity and/or the assignment of the sequence needs to be verified

The newly obtained sequences were aligned with those of other hypotrichs and four euplotids (outgroup) downloaded from GenBank (see Figs. 1, 2, 3, 4 for accession numbers). Sequences were aligned using MAFFT (FFT-NS-1 method) (Katoh and Standley 2013). The primers were manually trimmed using BioEdit v.7.0 (Hall 1999). The final alignment of SSU rDNA, ITS1-5.8S rDNA-ITS2, LSU rDNA, concatenated (SSU rDNA-ITS1-5.8S rDNA-ITS2-LSU rDNA), and COI sequences used for phylogenetic analyses were 1690, 577, 1853, 4229, and 478 base pairs long, respectively.

The best-fit models for maximum likelihood (ML) and Bayesian inference (BI) analyses were calculated by ModelFinder under Bayesian Information Criterion (BIC) (for details of models used, see Table 1). ML and BI analyses were performed according to Wang et al. (2022b). Trees were visualized with SeaView v.4.6.1 (Gouy et al. 2010) and MEGA 11 (Tamura et al. 2021). The standard of bootstrap values and Bayesian probabilities followed Hillis and Bull (1993) and Alfaro et al. (2003), respectively.

**Table 1:** Selected models used for phylogenetic analyses

	Region	IQ-TREE	MrBayes
Single sequence	SSU rDNA	TIM2 + F + R3	GTR + F + I + G4
	ITS1-5.8 rDNA-ITS2	SYM + I + G4	SYM + I + G4
	LSU rDNA	GTR + F + R3	GTR + F + I + G4
	COI	GTR + F + I + G4	GTR + F + I + G4
Concatenated sequence	Three genes	GTR + F + I + G4	GTR + F + I + G4

### Topology testing

To assess the monophyly or sister relationship of target taxa, the unbiased (AU) test (Shimodaira and Goldman 2002) was carried out by IQ-TREE v2.0. All tests performed 10,000 re-samplings using the RELL method.

## Results

### Systematics of the family Strongylidiidae (Figs. 1A–E, 2A, B, 3, 4, 5A, B, 6A, B, 7)

**Fig. 5:**
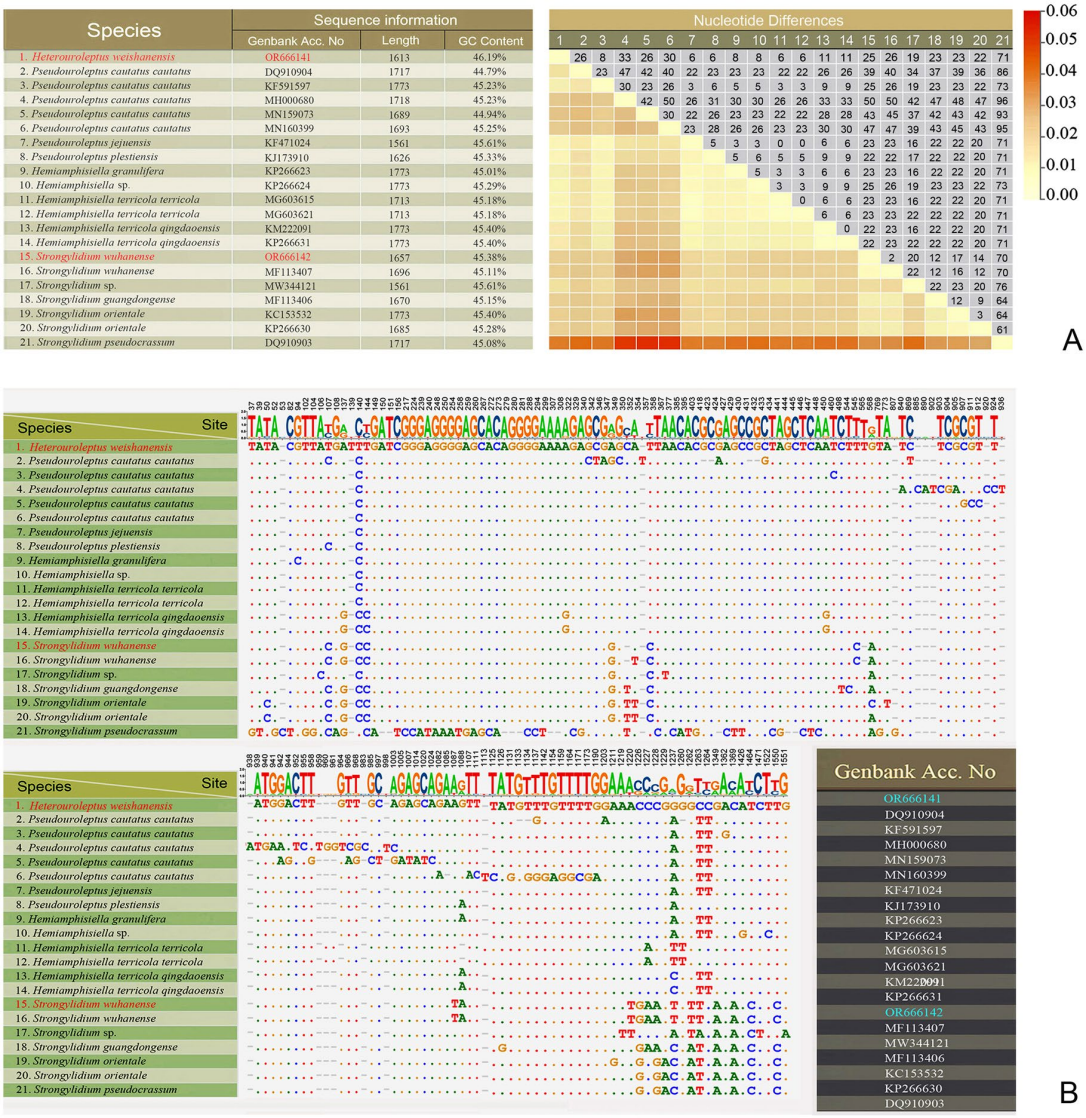
Nucleotide differences between strongylidiids based on SSU rDNA sequences. Lower left values of table **A** indicate the nucleotide differences, the upper right numbers are numbers of nucleotide differences. The numbers in the header **B** indicate the unmatched site positions. Newly obtained sequences are in red

**Fig. 6:**
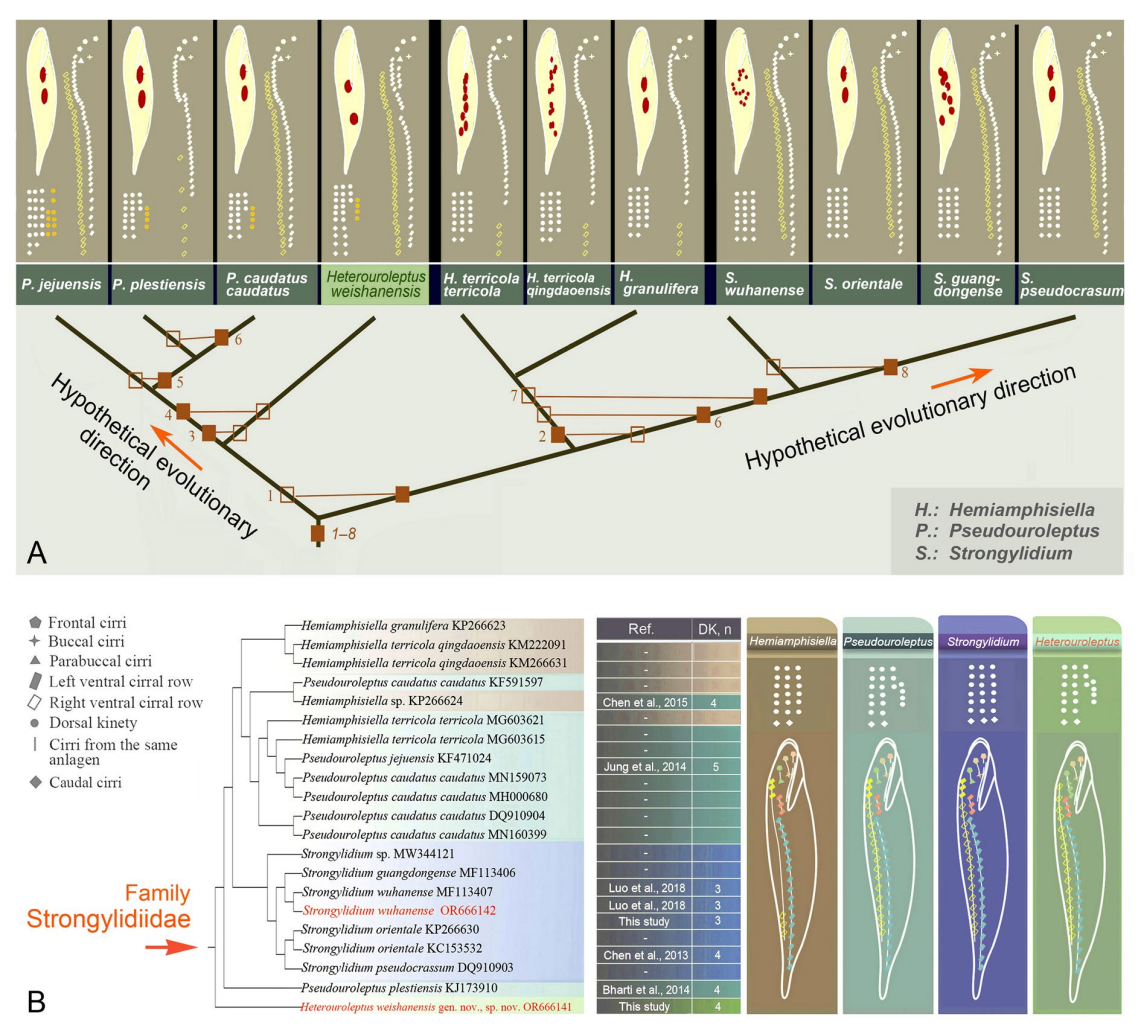
**A** Hypothetical systematic relationships within Strongylidiidae based on molecular and morphological data (for definitions of 1–8 see Table 5). **B** The morphogenetic information of four genera within Strongylidiidae mapped onto the SSU rDNA tree (branch in left was formed by MEGE 11 with Topology Only tool). Cirri originating from the same anlagen are shown in the same color. The right table shows the sequences are provided in the reference with corresponding taxonomic data. “−” indicates sequences that were uploaded directly and no corresponding morphological information was provided. *DK* *n* dorsal kineties number, *Ref* references

**Fig. 7:**
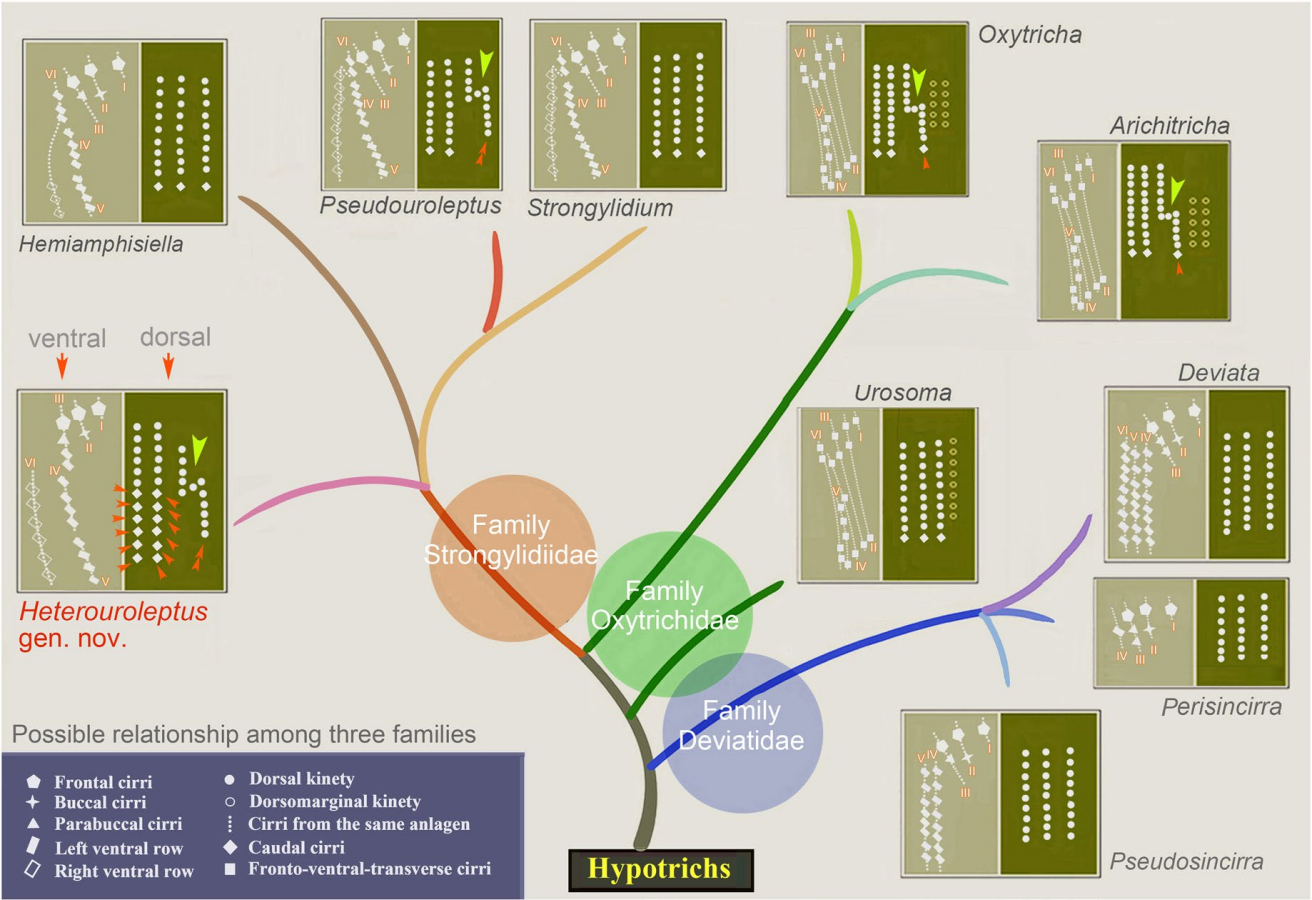
The morphogenetic information of strongylidiids, including *Heterouroleptus* gen. nov. and closely related genera, based on the SSU rDNA tree. Red arrowheads and red double arrowheads indicate the caudal cirri and dorsal kinety 4 formed by fragmentation of dorsal kinety 3, respectively; green arrowheads show the fragmentation of dorsal kinety 3. I–VI, frontal–ventral–transverse cirral anlagen I–VI

Phylogenetic trees generated from ML and BI analyses exhibited similar topologies, therefore, only the ML trees are presented here with nodal support values from both algorithms (Figs. 1, 2, 3, 4).

According to the SSU rDNA tree (Fig. 1, with *Apodiophrys ovalis* GU477634, *Diophrys scutum* JF694040, *Paradiophrys zhangi* FJ870076, and *Uronychia multicirrus* EU267929 as the outgroup), the family Strongylidiidae is monophyletic (100% ML/1.00 BI). *H. weishanensis* gen. nov., sp. nov. is sister to a clade that includes all other strongylidiids for which molecular data are available. Within this clade, *Pseudouroleptus plestiensis* (KJ173910) is sister to a clade that comprises two large subclades: in one, *Pseudouroleptus* and *Hemiamphisiella* are interdigitated with low ML support (68%) although this is incongruent with the topology of the BI tree; in the other large subclade, all *Strongylidium* species form a clade with nearly full support (98% ML/1.00 BI). Within the latter, the newly sequenced *S. wuhanense* (Weishan population, for details see Supplementary Fig. S1; Supplementary Table S1) clusters with the type population (Wuhan population) with full support and these two have only two nucleotide differences (Fig. 5). The possible evolutionary direction of *Heterouroleptus* gen. nov., other strongylidiid genera (*Hemiamphisiella*, *Pseudouroleptus*, and *Strongylidium*), and other related genera are shown in Figs. 6, 7.

The phylogenetic trees inferred from ITS1-5.8S rDNA-ITS2, LSU rDNA, and concatenated SSU rDNA, ITS1-5.8S rDNA-ITS2 and LSU rDNA are composed of 46, 43, and 46 sequences, respectively. In these three trees, all strongylidiids (except *Hemiamphisiella terricola qingdaoensis* KP266649 in the ITS1-5.8S rDNA-ITS2 tree and *Pseudouroleptus caudatus caudatus* KP266664 in the LSU rDNA tree, both of which are probably misidentified cluster in a large clade with high to full support (ITS1-5.8S rDNA-ITS2 tree, 98% ML/1.00 BI; LSU rDNA tree, 100% ML/1.00 BI; concatenated tree, 100% ML/1.00 BI). Within this clade, *H. weishanensis* gen. nov., sp. nov. is sister to a subclade comprising all the other strongylidiids, which is consistent with the SSU rDNA tree (Fig. 2).

A total of 47 sequences were used for phylogenetic analysis based on COI gene sequence data (Fig. 4), with *Uronychia setigera* (MG594868), *Uronychia binucleata* (MG594869), *Diophrys scutum* (MG594860), and *Diophrys oligothrix* (MG594865) as the outgroup. In this tree, all strongylidiids cluster within a clade with low to moderate support (63% ML/0.89 BI), but with *Quadristicha setigera* (MG594885) included.

## Taxonomy

### Diatirostomata Paiva, 2020

#### Strongylidiidae Fauré-Fremiet, 1961

##### *Heterouroleptus* gen. nov.

Diagnosis: Strongylidiids with dorsal kinety (DK) 4 fragmented from DK3-anlage; caudal cirri arranged in two rows, located at end of dorsal kineties 1, 2, respectively; left ventral cirral row composed of cirri from frontal–ventral–transverse cirral anlagen (FVTA) III (anterior portion), IV (middle portion), and V (rear portion); right ventral cirral row formed from entire FVTA VI.

Etymology: Composite of the Greek prefix *hetero* + (different) and the generic name *Uroleptus*. This alludes to the fact that the new genus resembles *Uroleptus* but differs from the latter by having a short (vs. long) tail.

Type species: *Heterouroleptus weishanensis* sp. nov.

Species assignable: The type species only.

### *Heterouroleptus weishanensis* sp. nov.

Diagnosis: Cell size 115–240 × 55–120 μm in vivo; cell fusiform with a short tail; contractile vacuole positioned 40–50% down length of cell near left cell margin; cortical granules colorless, globular, 0.8–2.0 μm in diameter; adoral zone composed of about 51 membranelles; one parabuccal, one or no post-peristomial ventral cirrus; left and right ventral cirral row with 31–56 and 31–57 cirri, respectively; left and right marginal row with 28–50 and 30–62 cirri, respectively; four dorsal kineties, with DK3 terminated posteriorly at about 1/3 of cell length; two long caudal cirral rows, located at end of dorsal kineties 1, 2, comprising 6–26 and 10–25 caudal cirri, respectively; two macronuclear nodules, two to four micronuclei; freshwater habitat.

**Fig. 8:**
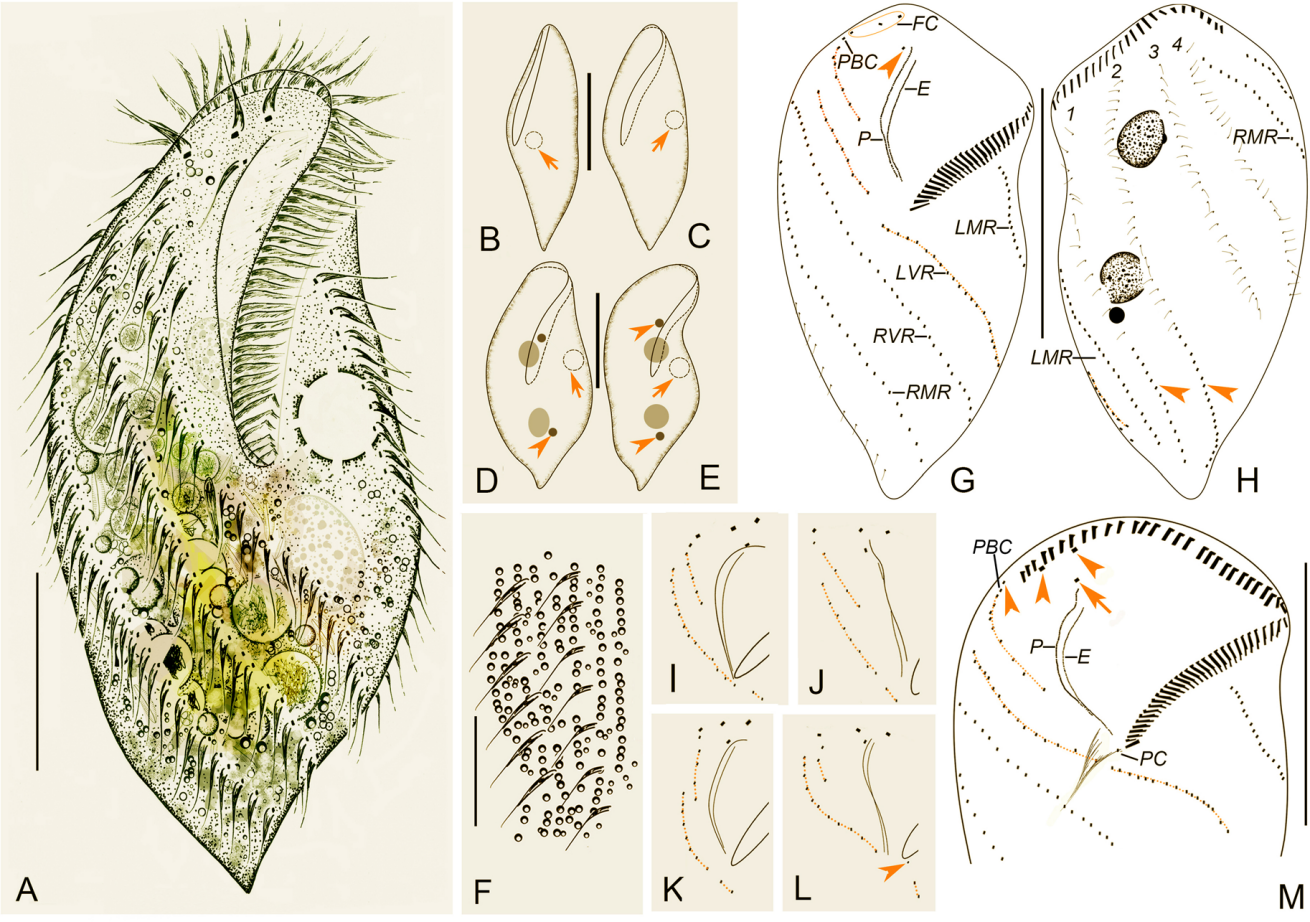
Morphology and infraciliature of *Heterouroleptus weishanensis* gen. nov., sp. nov. from life (**A**–**F**) and after protargol impregnation (**G**–**M**). **A** Ventral view of a representative individual. **B**–**E** Ventral (**C**–**E**) and lateral–ventral (**B**) views of cell with different shapes, arrows indicate the contractile vacuole, arrowheads in **D**, **E** show the micronuclei. **F** The arrangement of cortical granules on ventral side. **G**, **H** Ventral (**G**) and dorsal (**H**) views of the holotype specimen, showing the ciliary pattern and nuclear apparatus, arrowheads in **G** and **H** show the buccal and caudal cirri, respectively, dashed lines denote left ventral cirral row. **I**–**L** The different arrangements of left ventral cirral row (dashed lines) in the oral region, arrowhead in **L** shows the post-peristomial ventral cirrus. **M** Ventral view of the oral region, arrow and arrowheads indicate buccal cirrus and frontal cirri, respectively. 1–4, dorsal kineties 1–4; *E* endoral, *FC* frontal cirri, *LMR* left marginal row, *LVR* left ventral cirral row, *P* paroral, *PBC* parabuccal cirrus, *PC* post-peristomial ventral cirrus, *RMR* right marginal row, *RVR* right ventral cirral row. Bars: 20 μm (**F**), 50 μm (**A**, **B**–**E**, **G**, **H**, **M**)

Type material: One protargol slide (No. SWY2020062901/1) containing the holotype specimen (Fig. 8G, H) circled with black ink on the undersurface of the slide, and twelve paratype slides (Nos. SWY2020062901/2–13), have been deposited in the Laboratory of Protozoology, Ocean University of China, China.

Type locality: a freshwater aquaculture pond in Lake Weishan Wetland (34°46′24.2′′ N; 117°09′42.1′′ E), north-east China.

Etymology: The species-group name “*weishanensis*” refers to the area (Weishan) where the original population was collected.

### Morphology (Figs. 8A–M, 9A–P***; ***Table 2***)***

**Fig. 9:**
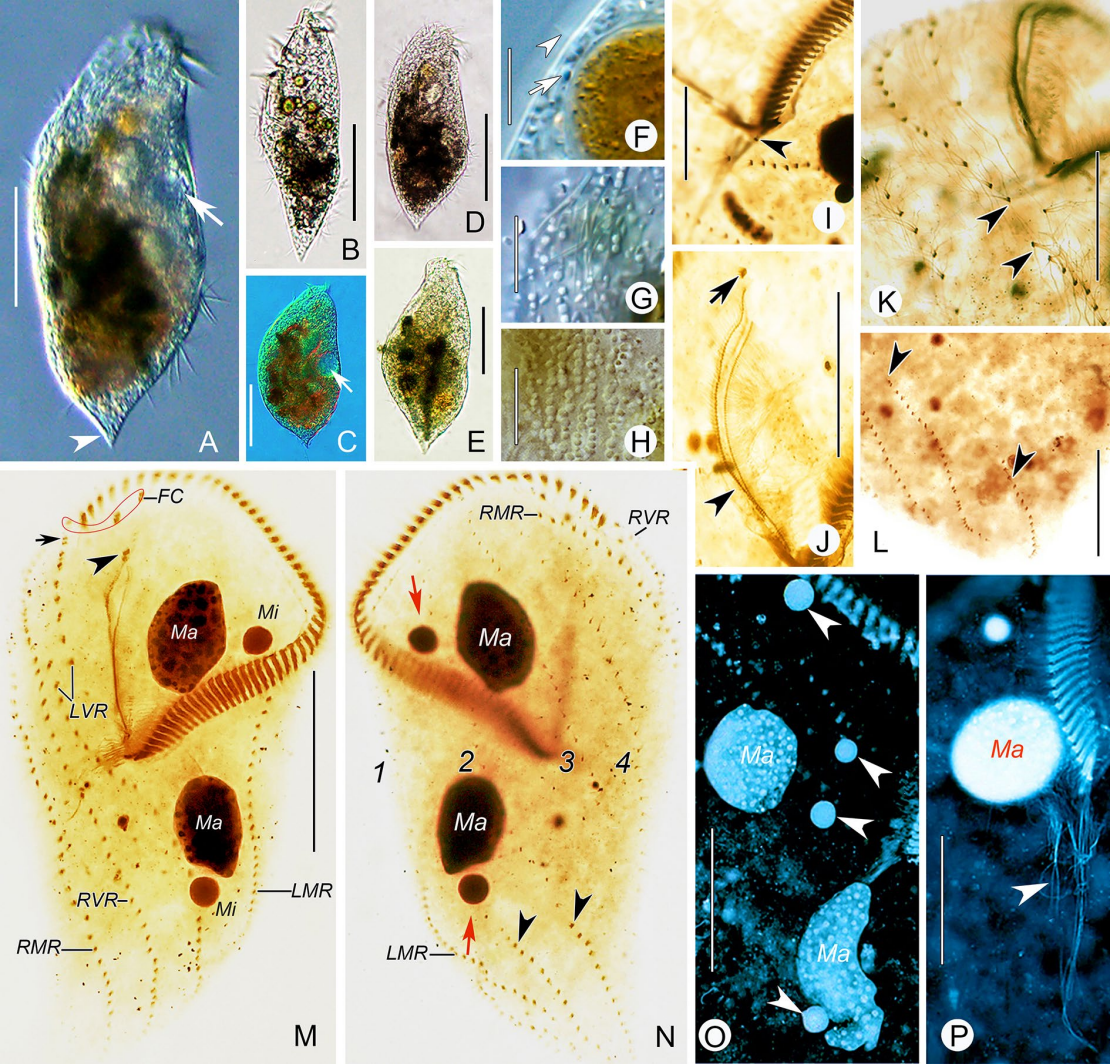
Photomicrographs of *Heterouroleptus weishanensis* gen. nov., sp. nov. from life **A**–**H** and after protargol impregnation (**I**–**P**). **B**, **D**, bright field; **A**, **C**, **E**–**H**, differential interference contrast. **A**–**E** Ventral view of a representative cell (**A**) and different individuals (**B**–**E**), arrow in (**A**, **C**) indicates contractile vacuole, arrowhead in **A** shows the tail. **F**–**H** The arrangement of cortical granules on the lateral (**F**), ventral (**G**), and dorsal (**H**) side, arrow and arrowhead in **F** indicate the cortical granules and the dorsal bristle, respectively. **I** Arrowhead indicates the post-peristomial ventral cirrus. **J** Arrow and arrowhead show the buccal cirrus and undulating membranes, respectively. **K** Arrowheads indicate the left ventral cirral row. **L** Arrowheads show the caudal cirri at the end of dorsal kineties 1, 2. **M**, **N** Ventral (**M**) and dorsal (**N**) view of a representative specimen, to show the infraciliature and nuclear apparatus, arrow and arrowhead in (**M**) demonstrate parabuccal and buccal cirrus, respectively, arrows and arrowheads in (**N**) show the micronuclei and the caudal cirri, respectively. **O**, **P** Adjusted by the invert function in Photoshop, (**O**) showing the macronuclear nodules and micronuclei (arrowheads), arrowhead in (**P**) denotes the pharyngeal fibers. 1–4, dorsal kineties 1–4, *FC* frontal cirri, *LMR* left marginal row, *LVR* left ventral cirral row, *Ma* macronuclear nodules, *Mi* micronuclei, *RMR* right marginal row, *RVR* right ventral cirral row. Bars: 20 μm (**F**–**H**, **I**–**L**, **O**, **P**), 50 μm (**A**–**E**, **M**, **N**)

**Table 2:** Morphometric data of *Heterouroleptus weishanensis* gen. nov., sp. nov.

Character	HT	Min	Max	Mean	M	SD	CV	n
Length of cell in μm	98	81	175	116.9	108	18.1	15.4	30
Width of cell in μm	52	36	120	63.1	52	16.4	26.0	30
Cell length: width, ratio (percentage)	1.9	1.5	2.9	2.0	2.0	0.3	16.2	30
Length of AZM in μm	43	35	71	47.8	44	5.6	11.7	30
AZM: cell length, ratio (percentage)	43.9	30.2	52.8	42.3	43.2	5.3	12.5	30
Adoral membranelles, number	50	38	62	50.6	51	6.5	12.9	25
Frontal cirri, number	3	3	3	3.0	3	0	0	25
Buccal cirrus, number	1	1	1	1.0	1	0	0	30
Parabuccal cirrus, number	1	1	1	1.0	1	0	0	25
Post-peristomial ventral cirrus, number	0	0	1	0.3	0	0.4	18.2	20
Cirral rows, number	4	4	4	4.0	4	0	0	25
Left ventral cirri, number	47	31	56	39.2	38	5.1	13.0	25
Right ventral cirri, number	47	31	57	45.9	48	8.6	18.8	18
Left marginal cirri, number	40	28	50	39.1	37	7.2	18.5	20
Right marginal cirri, number	47	30	62	49.3	50	8.7	17.7	21
Dorsal kineties, number	4	4	4	4.0	4	0	0	18
Dikinetids in DK1, number	14	9	19	14.7	15	2.8	18.8	18
Dikinetids in DK2, number	24	16	27	20.4	20	2.7	13.0	18
Dikinetids in DK3, number	26	18	34	24.9	25	4.7	18.7	18
Dikinetids in DK4, number	38	18	38	31.1	33	5.1	16.4	18
Caudal cirri, number	34	22	40	31.8	33	5.8	18.2	17
Caudal cirri after DK1, number	14	6	26	14.8	15	4.2	28.6	17
Caudal cirri after DK2, number	20	10	25	17.1	16	4.4	25.9	17
Macronuclear nodules, number	2	2	2	2.0	2	0	0	25
Length of anterior macronuclear nodule in μm	12	10	20	15.2	15	3.0	20.0	20
Width of anterior macronuclear nodule in μm	9	8	13	9.5	9	1.3	13.6	20
Length of posterior macronuclear nodule in μm	12	10	16	13.1	13	1.7	12.8	17
Width of posterior macronuclear nodule in μm	11	7	12	9.5	10	1.1	12.1	17
Micronuclei, number	2	2	4	2.5	2	0.8	32.2	20
Length of micronuclei in μm	2.8	2.3	4	3.5	3.6	0.4	12.4	20

All data are based on protargol-impregnated specimens

*AZM* adoral zone of membranelles, *CV* coefficient of variation in %, *DK1–4* dorsal kineties 1–4, *HT* holotype, *M* Median, *Max* maximum, *Mean* arithmetic mean, *Min* minimum, *n* number of cells measured, *SD* standard deviation

Cell 115–240 × 55–120 μm in vivo (*n* = 12), ratio of length to width ranging 1.9–2.8:1 (on average 2.3:1); in protargol-stained specimens, cell 81–175 × 36–120 μm (117 × 63 μm on average), with length: width ratio about 2.0:1 (range 1.5–2.9:1). Cell flexible and slightly contractile, outline more or less fusiform, narrowly rounded anteriorly and tapered posteriorly to form a short tail (Figs. 8A–E, 9A–E). Cortical granules colorless, globular, 0.8–2.0 μm in diameter, densely distributed along cirral rows and dorsal kineties throughout entire cell (Figs. 8F, 9F–H). Cytoplasm colorless, filled with lipid droplets (approximately 1–3 μm) and ingested algae of variable size, with algae giving cells a yellow-greenish or greenish appearance (Figs. 8A, 9A–E). Single contractile vacuole 14–17 μm across in diastole, located 40–50% down length of cell, close to left cell margin; collecting canals not observed (Figs. 8A–E, 9A, C). Nuclear apparatus situated left of midline, comprised of two ellipsoidal macronuclear nodules (17–22 × 14–16 μm in vivo), and two to four micronuclei, spherical to ellipsoidal, about 5 μm across, closely associated with the macronuclear nodules (Figs. 8D, E, H, 9N, O). Locomotion usually by moderately rapid swimming while rotating clockwise about main cell axis.

Adoral zone occupies nearly 45% of cell length in vivo, 30–53% (42% on average) after protargol impregnation, composed of 38–62 (on average 51) membranelles, curving across anterior cell margin and extending conspicuously down right side of cell (Figs. 8A–E, G, H, 9A, M; Table 2). Paroral longer than endoral, each consisting of a single row of dikinetids, distinctly curved, not optically intersecting in most specimens (intersecting in two out of 25 specimens) (Figs. 8G, I–M, 9J, M). Three clearly differentiated frontal cirri behind distal portion of adoral zone; single buccal cirrus located right of anterior end, or anterior of paroral; one parabuccal cirral row (III/2) located behind right frontal cirrus (Figs. 8A, G, I–M, 9J, M). Post-peristomial ventral cirrus usually absent (present in five out of 20 investigated specimens, located behind proximal end of adoral zone) (Figs. 8L, M, 9I). Two ventral cirral rows. Left ventral cirral row (LVR) with 31–56 cirri, usually consisting of three parts; the first part terminates behind parabuccal cirrus; the second part commences at right of posterior-most cirri of the first part; the third part commences at level of proximal end of adoral zone of membranelles (AZM) and terminates near end of cell on dorsal side; a gap is present between the second and third parts (Figs. 8A, G, I–M, 9K, M). Right ventral cirral row (RVR) with 31–57 cirri, commences dorsally near distal end of AZM, extends ventrally, and terminates near posterior end of cell left margin on dorsal side (Figs. 8A, G, H, 9M, N). One left and one right marginal row (LMR and RMR), both nearly reach posterior end of cell, composed of 28–50 and 30–62 cirri, respectively; LMR commences on ventral side and terminates on dorsal side; RMR commences on dorsal side and terminates on ventral side (Figs. 8A, G, H, M, 9M, N). Cilia of cirral rows about 14–16 μm long in vivo.

Invariably four dorsal kineties (DK) composed of 9–19, 16–27, 18–34, and 18–38 dikinetids, respectively. Dorsal bristles about 3 μm long and closely spaced. DK1–3 commence near anterior cell end and terminate in posterior 35% of cell; DK4 commences at anterior end of cell, extends onto ventral surface and terminates at posterior end of cell (Figs. 8G, H, 9N). Caudal cirri form two rows, located at ends of DK1, 2; caudal cirral row 1 with 6–26 cirri, row 2 with 10–25 cirri, 22–40 caudal cirri in total (Figs. 8H, 9L, N).

### Morphogenesis (Figs. 10A–L, 11A–O)

**Fig. 10:**
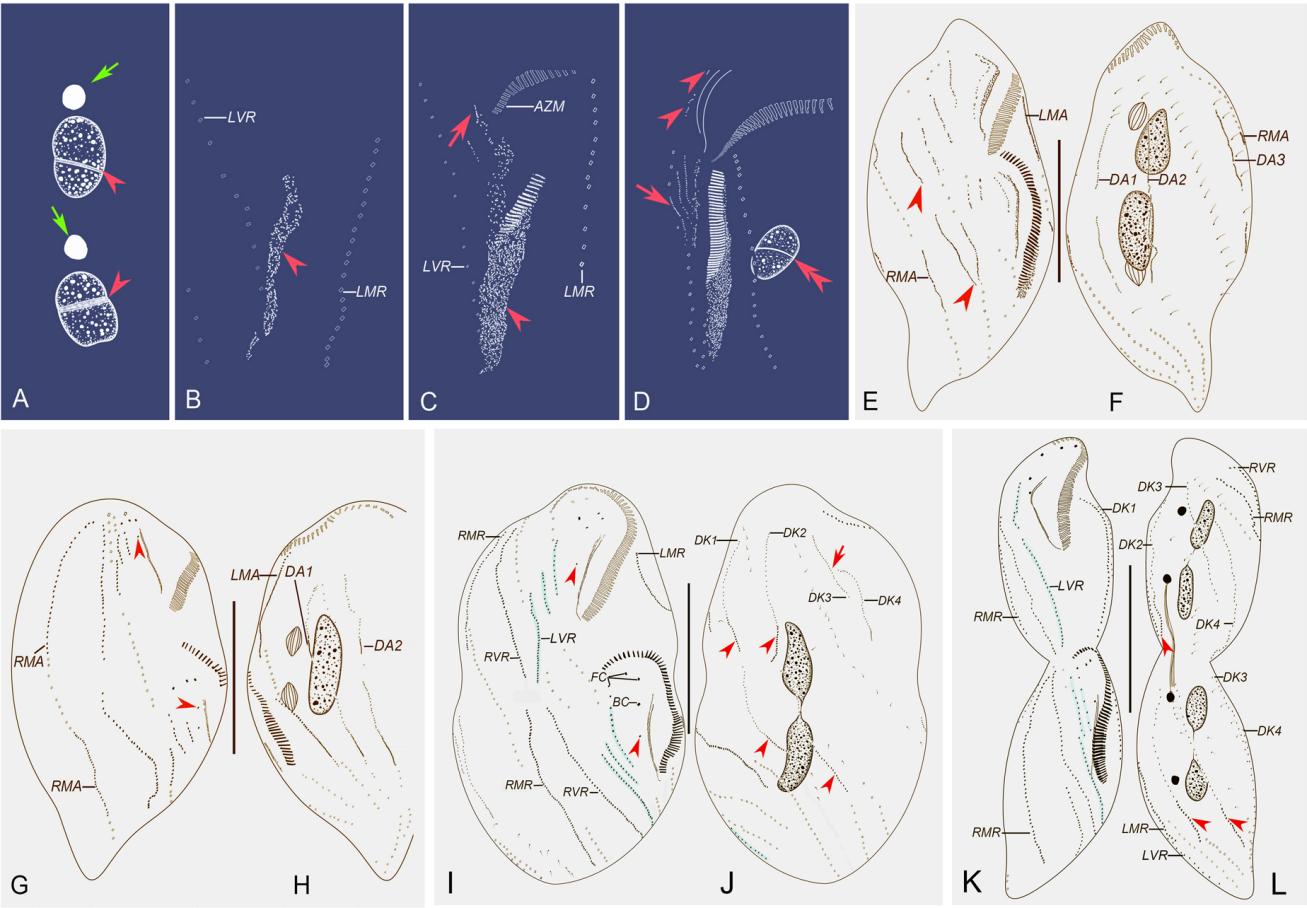
Morphogenetic process of *Heterouroleptus weishanensis* gen. nov., sp. nov. after protargol impregnation. **A** Nuclear apparatus of a very early divider, showing replication band of macronuclear nodules (arrowheads) and micronuclei (arrows). **B** Oral primordium of the opisthe (arrowhead). **C** Ventral view of an early divider, to show developed oral primordia (arrowhead), partial undulating membrane anlage, and frontal–ventral–transverse cirral anlagen (arrow). **D** Ventral view of an early divider, arrow shows the frontal–ventral–transverse cirral anlagen, arrowheads indicate the dedifferentiation of buccal cirrus and formation of FVTA II, and double-arrowhead denotes the replication band of macronuclear nodules. **E**, **F** Ventral (**E**) and dorsal (**F**) views of an early-middle divider, arrowheads mark the frontal–ventral–transverse cirral anlage VI (anlage for the right ventral cirral row) formed de novo in the proter and opisthe. **G**, **H** Ventral (**G**) and dorsal (**H**) view of a middle divider, arrowheads show the buccal cirri. **I**, **J** Ventral (**I**) and dorsal (**J**) of the same late divider, arrowheads in (**I**) show the post-peristomial ventral cirri in the proter and opisthe, arrow and arrowheads in (**J**) indicate the fragmentation of dorsal kinety 3 and caudal cirri, respectively. **K**, **L** Ventral (**K**) and dorsal (**L**) views of the same very late divider, arrowheads in (**L**) show the caudal cirri. Left ventral cirral row is shaded blue. *AZM* adoral zone of membranelles, *BC* buccal cirri, *DK1–4* dorsal kineties 1–4, *DA1–3* dorsal kineties anlagen 1–3, *FC* frontal cirri, *LMA* left marginal anlage, *LMR* left marginal row, *LVR* left ventral cirral row, *RMA* right marginal anlage, *RMR* right marginal row, *RVR* right ventral cirral row. Bars: 20 μm (**A**–**D**), 50 μm (**E**–**L**)

**Fig. 11:**
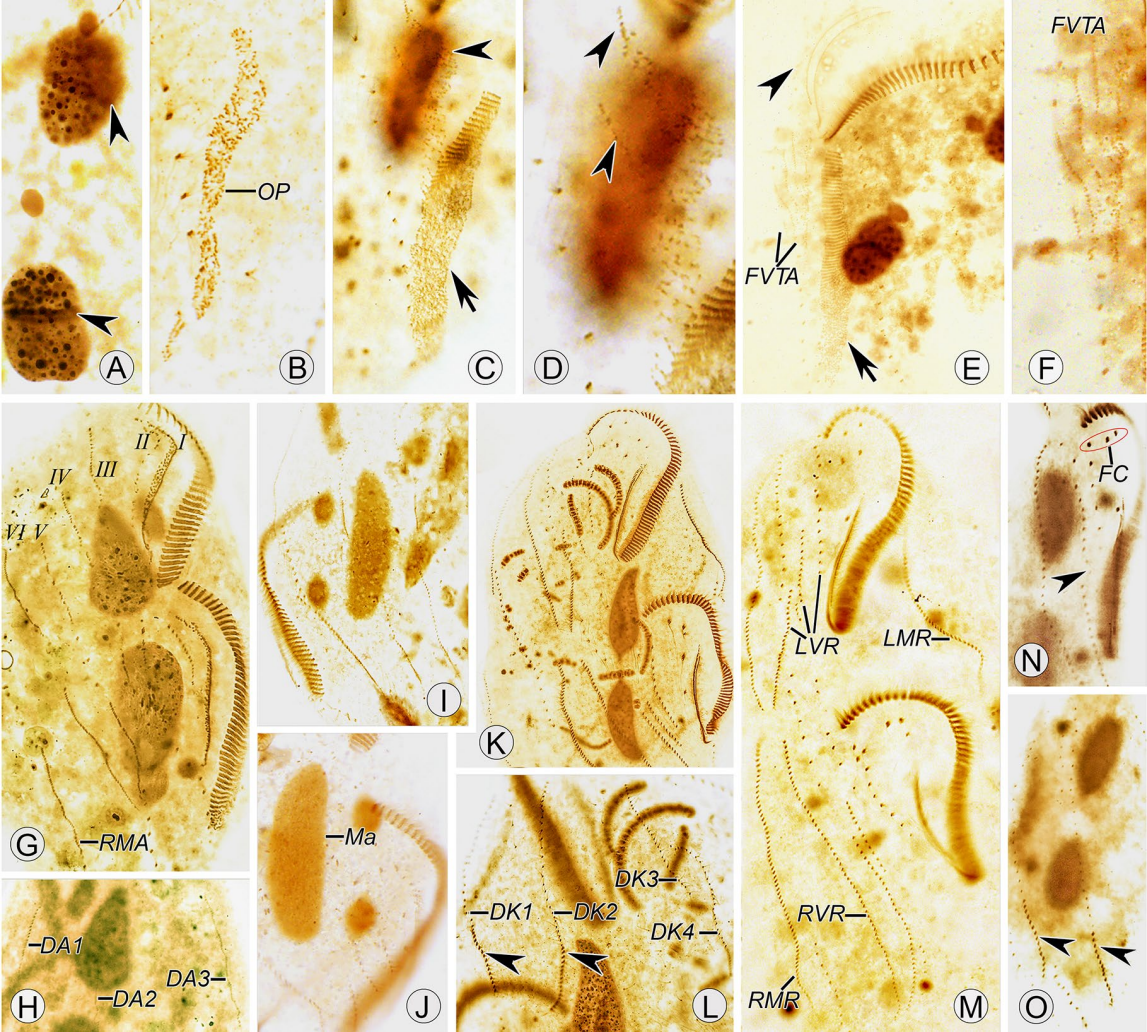
Morphogenetic process of *Heterouroleptus weishanensis* gen. nov., sp. nov. after protargol impregnation. **A** The replication band of macronuclear nodules (arrowheads). **B** Ventral view of an early divider, showing the appearance of the oral primordium of the opisthe. **C**, **D** Ventral view of the same early divider cell, arrow in **C** shows the differentiation of adoral zone of membranelles, arrowhead in **C**, **D** shows the frontal–ventral–transverse cirral anlagen. **E**, **F** Ventral view, arrowhead and arrow in **E** mark the formation of FVTA II and incomplete adoral zone of membranelles, respectively. **G**, **H** Ventral (**G**) and dorsal (**H**) views of a same early divider, showing the elongated frontal–ventral–transverse cirral anlagen and dorsal kineties anlagen. **I**, **J** Dorsal (**I**) and ventral (**J**) views of a middle divider. **K**, **L** Dorsal (**K**) and ventral (**L**) view of a middle divider, arrowheads in (**L**) denote the caudal cirri. **M** Ventral view of a very late divider. **N**, **O** Ventral (**N**) and dorsal (**O**) views of a very late divider, arrowhead in (**N**) denotes gap of left ventral cirral row and arrowheads in (**O**) denote the caudal cirri. I–VI, frontal–ventral–transverse cirral anlagen I–VI, *DA1–3* dorsal kineties anlagen 1–3, *DK1–4* dorsal kineties 1–4, *FC* frontal cirri, *FVTA* frontal–ventral–transverse cirral anlagen, *LMR* left marginal row, *LVR* left ventral cirral row, *Ma* macronuclear nodules, *OP* oral primordium, *RMA* right marginal anlage, *RMR* right marginal row, *RVR* right ventral cirral row. Bars: 10 μm (**B**, **D**), 20 μm (**A**, **C**, **E**, **H**–**J**, **L**, **N**, **O**), 50 μm (**G**, **K**, **M**)

#### Stomatogenesis and ventral ciliature

In the very early stage, the oral primordium of the opisthe develops de novo to the left of the posterior portion of the LVR (Figs. 10B, 11B). Subsequently, the oral primordium for the opisthe enlarges by proliferation of basal bodies, and several membranelles form in the anterior portion (Figs. 10C, 11C). Meanwhile, the undulating membranes anlage (UMA), that is frontal–ventral–transverse cirral anlage (FVTA) I, and FVTA II, for the opisthe appear to the right of the oral primordium (Figs. 10C, 11C, D). Later, in the proter, the buccal cirrus dedifferentiates and FVT II is formed; in the opisthe, FVTA III appears to the right of FVTA II, and FVTA IV forms within the parental left ventral cirral row (Figs. 10D, 11E, F). In the next stage, in the proter, the parental paroral begins to disintegrate and forms the UMA, and the buccal cirrus, parabuccal cirrus, and left ventral cirri dedifferentiate to form FVTA II, III, and IV, respectively. The old right ventral cirri take part in the formation of FVTA V to the right of which FVTA VI originates de novo in both proter and opisthe (Figs. 10E, 11G). From middle to late stages, the formation of the oral primordium for the opisthe is gradually completed from anterior to posterior, and the UMA for each daughter cell splits longitudinally into undulating membranes (Figs. 10G, I, 11K, M). During this time, the differentiation of the FVTA is almost finished: FVTA I differentiates into the left frontal cirrus; FVTA II generates the middle frontal cirrus and the buccal cirrus; FVTA III contributes the right frontal cirrus, parabuccal cirrus and the anterior segment of the LVR; FVTA IV gives rise to the median segment of the LVR; FVTA V differentiates into the posterior segment of the LVR; and FVTA VI generates the RVR (Figs. 10G, I, 11K, M). At the final stage, the paroral and endoral are separated, the ciliature is differentiated, the migration process is almost finished, the parental adoral zone is completely retained by the proter, and the parental marginal and ventral cirri are absorbed. The origin of the post-peristomial cirrus is not clear (Figs. 10K, 11N).

#### Formation of the marginal cirri, dorsal kineties, and caudal cirri

At the early-late stage, the marginal anlagen develop intrakinetally in both the proter and the opisthe (Figs. 10E, F, 11G). Both marginal anlagen then lengthen towards both ends and generate new cirri that replace the old structures (Figs. 10G–L, 11I, K, M, N).

In early-middle dividers, three thread-like dorsal kineties anlagen (DKA) appear. In both the opisthe and proter, DKA1 and DKA2 develop intrakinetally, and DKA3 emerges as a row of basal bodies that develop de novo to the right of parental dorsal kinety 4 (Figs. 10F, H, 11H). The DKA then stretch mainly posteriad and gradually replace the parental structures. In late dividers, DKA3 fragments to form two anlagen in the posterior region. Thus, four rows of dorsal kineties are formed in each daughter cell (Figs. 10J, L, 11L). During the morphogenetic process, the two caudal cirral rows are generated, one each at the end of DKA1 and 2 (Figs. 10J, L, 11L, O).

#### Division of nuclear apparatus

In the very early stage, a replication band is present in each macronuclear nodule (Figs. 10A, 11A). The division of the nuclear apparatus proceeds in the usual way for hypotrichs (Berger 2008), i.e., the two macronuclear nodules fuse into a single mass which subsequently makes successive amitotic divisions to produce the species-specific number of nodules in each filial product. The micronuclei divide mitotically (Figs. 10D, F, H, J, L, 11I, K).

## Discussion

### Phylogenetic analyses of the family Strongylidiidae

The family Strongylidiidae was erected by Fauré-Fremiet (1961), with *Strongylidium* as the type genus. Later, this family was treated as a junior synonym of Spirofilidae (Jankowski 2007; Lynn 2008; Lynn and Small 2002; Shi 1999b; Tuffrau and Fleury 1994). Recently, Luo et al. (2018) improved the diagnosis for Strongylidiidae and proposed that: (1) the mixed left ventral cirral row and the many shared morphological features are unlikely to be the product of convergent evolution but rather they share a common ancestor, which is supported by the phylogenetic analyses; (2) Strongylidiidae has a close relationship with Dorsomarginalia based on SSU rDNA sequence data. In the present study, all strongylidiids cluster together, then form a sister clade to the clade of dorsomarginalian species in SSU rDNA, ITS1-5.8S rDNA-ITS2, and concatenated trees. In the LSU rDNA tree, however, the deviatid and dorsomarginalian species cluster together, suggesting that the family Strongylidiidae is monophyletic, which is consistent with the findings of Luo et al. (2018).

The strongylidiid genus *Pseudouroleptus* was established by Hemberger (1985), with the soil ciliate *P. caudatus* Hemberger, 1985 as the type species. Subsequently, Foissner et al. (2002) split *P. caudatus* into two subspecies, *P. caudatus caudatus* Hemberger, 1985 and *P. caudatus namibiensis* Foissner et al., 2002, mainly based on the distinct difference in the number of ‘transverse cirri’, which are actually right ventral cirri according to Berger (2008). Berger (2008) transferred four *Pseudouroleptus* species into the new genus *Bistichella* Berger, 2008, i.e., *Bistichella buitkampi* (Foissner, 1982) Berger, 2008, *Bistichella procera* (Berger & Foissner, 1987) Berger, 2008, *Bistichella terrestris* (Hemberger, 1985) Berger, 2008, and *Bistichella humicola* (Gellért, 1956) Berger, 2008. Consequently, only the type species, *P. caudatus*, remained in the genus *Pseudouroleptus*. Since then, two more species, *Pseudouroleptus jejuensis* Jung et al., 2014 and *Pseudouroleptus plestiensis* Bharti et al., 2014, have been described (Bharti et al. 2014; Jung et al. 2014). All three *Pseudouroleptus* species have been described in detail including their ontogenetic processes and corresponding SSU rDNA sequence information (Berger 2008; Bharti et al. 2014; Chen et al. 2015; Jung et al. 2014; Song and Shao 2017). For *P. jejuensis* and *P. plestiensis*, data on other genetic markers (e.g., ITS1-5.8S rDNA-ITS2, LSU rDNA, and COI gene) remain unavailable.

Previous studies have shown that *Pseudouroleptus* invariably clusters with *Hemiamphisiella*, a strongylidiid genus with *Hemiamphisiella terricola terricola* (Foissner, 1988) Berger, 2008 as the type species. The other species and subspecies in this genus are *H. terricola qingdaoensis* (Song & Wilbert, 1989) Berger, 2008, *Hemiamphisiella granulifera* (Foissner, 1987) Foissner, 1988, and *Hemiamphisiella wilberti* (Foissner, 1982) Foissner, 1988 (Berger, 2008). Morphologically, *Pseudouroleptus* and *Hemiamphisiella* are similar in most features. However, they differ from each other in terms of the presence/absence of fragmentation of dorsal kinety 3; for example, this fragmentation is present in all species of *Pseudouroleptus*, whereas for *Hemiamphisiella* the fragmentation is possibly present in *H. terricola terricola* (four dorsal kineties are present in the type population, but only three dorsal kineties are present in other populations) and in *H. wilberti* (four dorsal kineties present) but absent in *H. granulifera* (Berger 2008). In addition, most *Pseudouroleptus* species can be distinguished from *Hemiamphisiella* species by the RVR being long and extending to near the posterior end of the cell (vs. RVR short and commencing from the middle or near posterior end of the cell). However, a short RVR (3–7 cirri) is also present in one *Pseudouroleptus* species, namely *P. plestiensis* (Bharti et al. 2014). Thus, both *Hemiamphisiella* and *Pseudouroleptus* show variable morphological characteristics (the presence/absence of fragmentation of dorsal kinety 3 in *Hemiamphisiella* and the length of RVR in *Pseudouroleptus*), which indicate that these two genera may need to be further divided.

Consistent with previous studies, *Pseudouroleptus* is intermingled with *Hemiamphisiella* based on SSU rDNA sequence data presented here. It should be noted that the sequence *Pseudouroleptus caudatus caudatus* (KF591597), provided by Chen et al. (2015) with a detailed morphological description, has 23–30 nucleotide differences from the other four sequences under the name *P. caudatus caudatus* (DQ910904, MH000680, MN159073, MN160399), morphological data for which are unavailable. Therefore, the four above-mentioned populations of *P. caudatus caudatus* are likely misidentified and probably represent a separate species, thus needing further investigation. Furthermore, two sequences under the name of *Hemiamphisiella terricola terricola* (MG603621 and MG603615) have no nucleotide difference with *P. jejuensis* (KF471024). The two former sequences lack morphological data or vouchered specimens, whereas the latter was described in detail by Jung et al. (2014). Thus, it is possible that the sequences of *Hemiamphisiella terricola terricola* (MG603621 and MG603615) were misidentified and are conspecific with *P. jejuensis*.

It is unfortunate that currently there is only one ITS1-5.8S rDNA-ITS2, and one LSU rDNA, sequence available for the genus *Pseudouroleptus*. We cannot therefore give more insight into the systematic position of *Pseudouroleptus* based on these two nuclear genes*.* It is noteworthy that *P. caudatus caudatus* (KP266664) branches off from the strongylidiid clade in the LSU rDNA tree, which indicates this sequence is misidentified. *Hemiamphisiella* sequences cluster together in the LSU rDNA and concatenated rDNA trees with near full support, but *Hemiamphisiella terricola qingdaoensis* (KP266649) branches off from the strongylidiid clade in the ITS1-5.8S rDNA-ITS2 tree, which indicates this sequence needs to be verified.

Based on the SSU rDNA tree, *Pseudouroleptus plestiensis* (KJ173910) falls outside the *Pseudouroleptus* clade. AU tests indicate the sequence of *P. plestiensis* influences the monophyly of *Pseudouroleptus*: (1) by enforcing all *Pseudouroleptus* species, the monophyly of *Pseudouroleptus* is rejected (*p* = 0.048) (Table 3); (2) by enforcing all *Pseudouroleptus* species excluding *Pseudouroleptus plestiensis* (KJ173910), the monophyly of *Pseudouroleptus* has a high reliability (*p* = 0.607) (Table 3). A detailed morphological characterization of *Pseudouroleptus plestiensis* (KJ173910) was provided by Bharti et al. (2014), and its short right ventral cirral row distinguishes it from all the other *Pseudouroleptus* species. As discussed above, combining morphological and molecular phylogenetic information, we speculate that *Pseudouroleptus plestiensis* may represent a separate genus which could be diagnosed by having fragmentation of dorsal kinety 3, and a short right ventral cirral row. Furthermore, the AU tests do not refute the monophyly of *Hemiamphisiella* in the four nuclear gene trees (*p* = 0.29–0.608) (Table 3). The close relationship between *Hemiamphisiella* and *Pseudouroleptus* is also not rejected in SSU rDNA, ITS1-5.8S rDNA-ITS2, and concatenated trees. In this case, we agree with Berger (2008) that some *Hemiamphisiella* species were perhaps misclassified due to the absence of morphogenetic data on the dorsal ciliature. The genera *Hemiamphisiella* and *Pseudouroleptus* share similar ciliature patterns, and each forms a distinct clade in the ITS1-5.8S rDNA-ITS2 and concatenated trees (84% ML/ 0.92 BI in the former, 66% ML/ 0.99 BI in the latter). Therefore, we regard *Hemiamphisiella* and *Pseudouroleptus* as separate genera, indicating that compared to SSU rDNA alone, the addition of multiple gene markers, e.g., ITS1-5.8S rDNA-ITS2, LSU rDNA, in phylogenetic analyses can help to better understand the evolutionary relationship between *Hemiamphisiella* and *Pseudouroleptus*.

**Table 3:** Approximately Unbiased test (AU test) results based on the single-gene and concatenated rDNA data

Topology constraints	AU value (*p*)				
	SSU rDNA	LSU rDNA	ITS1-5.8S rDNA-ITS2	Concatenated rDNA	COI
Best scoring unconstrained tree	0.607	0.469	0.576	0.449	0.552
*Hemiamphisiella*	0.576	0.557	0.29	0.496	0.535
*Hemiamphisiella* (excluding MG603621 and MG603615)	0.608	–	–	–	–
*Pseudouroleptus*	**0.048**	–	–	–	–
*Pseudouroleptus* (including MG603621 and MG603615)	0.0467	–	–	–	–
*Pseudouroleptus* (KJ173910 excluded)	0.607	–	–	–	–
*Strongylidium*	0.607	0.433	0.596	0.516	0.528
*Pseudouroleptus* + *Hemiamphisiella*	0.576	**9.82e-162**	0.289	0.467	0.298
*Pseudouroleptus* + *Hemiamphisiella* (KJ173910 excluded)	0.595	–	–	–	–

The column of topology constraints refers to proposed taxonomic groups that were tested for monophyly through the AU test. Rejected hypotheses (*p* < 0.05) are highlighted in bold

Phylogenetic analysis of COI gene sequence data showed that the family Strongylidiidae is non-monophyletic, as *Quadristicha setigera* clusters within it, although with very low support (63% ML/0.89 BI) (Fig. 4). The AU tests do not refute the close relationship between *Hemiamphisiella* and *Pseudouroleptus* (*p* = 0.298) (Table 3). Furthermore, the COI gene shows higher resolution than the SSU rDNA in terms of nucleotides differences, with *H. weishanensis* sp. nov. gen. nov. differing from other strongylidiids by 79–89 nucleotides, corresponding to 81.3–83.4% similarity, for COI (vs. 6–71 nucleotides, corresponding to 95.7–99.6% similarity for SSU rDNA). According to the COI tree, Strongylidiidae has a close relationship with gonostomatids, oxytrichids, and neokeronopsids, which is inconsistent with the nuclear rRNA trees. This may indicate that the COI gene shows low reliability of the interior branches at the higher level taxa, such as family or order. However, due to undersampling and the paucity of molecular data, it is too early to draw further conclusions based on this gene.

### Establishment of *Heterouroleptus* gen. nov. and comparison with closely related taxa (Table 4)

**Table 4:** Morphometrical, morphological, and morphogenetic comparisons between *Heterouroleptus*, *Pseudouroleptus* and *Hemiamphisiella* species

Character	1	2	3	4	5	6	7	8	9
Length	115–240	230–320	180–330	90–160	220–300	140–200	220–300	170–240	150–200
Width	55–120	45–65	40–60	20–40	35–55	25–40	50–65	25–45	150–200
CG	Present	Present	Present	Absent	Present	Present	Absent	Present	Absent
AZM	38–62	54–67	33 –51	26–32	52–62	23–37	42–53	37–52	28–35
LVC	31–56	60–77	50–67	27–40	55–72	28–51	55–72	54–69	37–46
RVC	31–57	51–65	20–31	3–7	46–58	3–4	15–27	8–14	2–3
LMR	28–50	56–72	44–61	26–45	53–66	34–57	50–71	50–71	28–39
RMR	30–62	57–76	47–70	31–47	53–66	35–53	52–75	57–71	35–41
CC	22–40	3–6	1–4	1–2	4–7	Probably 2	3?		3
DK	4	4	4	4	5	3	4	4	3
Ma, n	2	2	2	2	2	2	2	25–35	10–16
Mi, n	2–4	2–7	1–4	2–4	2–4	1–5	2–3	1–6^#^	1–5
Habitat	Freshwater	Soil	Terrestrial	Soil	Soil	Terrestrial	Limnetic	Soil	Terrestrial
Origin of LVR	A	B	–	B	B	–	–	B	–
Origin of RVR	The entire FVTA VI	C	–	C	C	–	–	C	–
RVR length*/**	Long	Long	Short	Short	Long	–	Short	Short	Short
DKF	Present	Present	Present	Present	Present	–	–	Absent	Absent
Data source	This study	Chen et al. (2015)	Foissner et al. (2002)	Bharti et al. (2014)	Jung et al. (2014)	Berger (2008)	Berger (2008)	Foissner (1987)	Song and Wilbert (1989)

Length and width according to the cell length and width in vivo (μm). 1, *Heterouroleptus weishanensis* gen. nov., sp. nov.; 2, *Pseudouroleptus caudatus caudatus*; 3, *Pseudouroleptus caudatus namibiensis*; 4, *Pseudouroleptus plestiensis*; 5, *Pseudouroleptus jejuensis*; 6, *Hemiamphisiella granulifera*; 7, *Hemiamphisiella wilberti*; 8, *Hemiamphisiella terricola terricola*; 9, *Hemiamphisiella terricola qingdaoensis*. A, the origin of LVR is formed by FVTA III (anterior portion), IV (middle portion), and V (rear portion), *AZM* adoral zone of membranelles, B, FVTA VI (anterior portion), IV (middle portion), and V (rear portion), C, posterior portion of FVTA VI; *CC* caudal cirri, *CG* cortical granules, *DK* dorsal kineties, *DKF* dorsal kineties fragmentation, *FVTA VI* frontal–ventral–transverse cirral anlage VI, *LMR* left marginal row, *LVC* left ventral cirri, *LVR* left ventral cirral row, *Ma* macronuclear nodules, *Mi* micronuclei, *RMR* right marginal row, *RVC* right ventral cirri, *RVR* right ventral cirral row, “*” represents the RVR commencing at the anterior of cell and extending to near the posterior end of cell, “**”, represents RVR commencing from the middle or the near the posterior end of cel, “#” data from Blatterer and Foissner (1988)

Based on the phylogenetic analysis discussed above, the clustering of *H. weishanensis* with strongylidiids and the nucleotide differences of the SSU rDNA (6–71 base pairs) between *H. weishanensis* and other strongylidiids (Fig. 5) support the establishment of *Heterouroleptus* as a new strongylidiid genus.

With respect to its infraciliature, that is, three clearly differentiated frontal cirri, the presence of buccal and caudal cirri, two more or less long ventral cirral rows, and the absence of pretransverse ventral cirri, transverse cirri, and dorsomarginal kineties, *Heterouroleptus* should be compared to three strongylidiid genera, i.e., *Pseudouroleptus*, *Hemiamphisiella*, and *Strongylidium* (Berger 2008; Luo et al. 2018).

Morphologically, *Heterouroleptus* gen. nov. is most similar to *Pseudouroleptus*, both of which have almost the same cirral pattern and dorsal kinety 3 fragmentation (Berger 2008; Bharti et al. 2014; Chen et al. 2015; Jung et al. 2014). However, these genera can be separated based on ontogenetic information (see next paragraph) and molecular data (6–33 base pairs difference in SSU rDNA). *Heterouroleptus* gen. nov. can be easily separated from *Hemiamphisiella* by having a long (vs. short in *Hemiamphisiella*) right ventral cirral row (Berger 2008; Eigner and Foissner 1994). *Heterouroleptus* gen. nov. differs from *Strongylidium* by having four dorsal kineties with dorsal kinety 4 fragmented from dorsal kinety 3 (vs. having only three dorsal kineties without fragmentation in *Strongylidium*) (Chen et al. 2013; Luo et al. 2018; Silva Paiva and Silva-Neto 2007; Sterki 1878).

Morphogenetically, *Heterouroleptus* gen. nov. can be separated from *Pseudouroleptus*, *Hemiamphisiella*, and *Strongylidium* as follows: (1) the LVR originates from FVTA III (anterior portion), IV (middle portion), and V (rear portion) in *Heterouroleptus* gen. nov., whereas the anterior, middle, and rear portion of the LVR originate from FVTA VI, IV, and V, respectively, in the latter three genera; (2) the RVR originates from the entire FVTA VI in *Heterouroleptus* gen. nov., whereas in the latter three genera, it originates from the rear portion of FVTA VI (Fig. 7) (Berger 2008; Bharti et al. 2014; Foissner et al. 2002; Hemberger 1985; Jung et al. 2014; Luo et al. 2018). The morphological and morphogenetic differences indicate that *H. weishanensis* can be distinguished from all known strongylidiids and should not be assigned to any existing genus (Table 4). Due to the novel morphogenetic characters of the LVR and RVR, the diagnosis of the family Strongylidiidae can be emended.

Improved diagnosis of Strongylidiidae: Non-dorsomarginalian hypotrichia with elongate cell usually distinctly narrowed posteriorly and slightly twisted about main cell axis. Three enlarged frontal cirri; one buccal cirrus; usually one cirrus (= cirrus III/2) left of anterior portion of left ventral cirral row; postoral ventral cirrus either present or absent, originates from FVTA IV; pretransverse ventral and transverse cirri absent; left ventral cirral row composed of cirri from three anlagen; right ventral cirral row originates from entire FVTA VI or posterior portion of VI; one marginal cirral row on each side; caudal cirri present; dorsal kinety fragmentation present or secondarily lost.

Type genus: *Strongylidium* Sterki, 1878.

Genera included: *Strongylidium* Sterki, 1878; *Hemiamphisiella* Foissner, 1988; *Pseudouroleptus* Hemberger, 1985; *Heterouroleptus* gen. nov.

### Hypothesis of evolutionary relationships within family Strongylidiidae (Fig. 6)

Based on morphological, morphogenetic, and phylogenetic data, the evolutionary relationships of taxa within the family Strongylidiidae are hypothesized. All strongylidiids share a distinct feature, that is, the LVR originates from three anlagen. In terms of the right ventral cirral row developing de novo to the right of the parental structure, all species of *Hemiamphisiella* and *Pseudouroleptus,* and *H. weishanensis* gen. nov., sp. nov., should be mentioned, and they group together in SSU rDNA tree. Although *Strongylidium* species also fall within this group, their parental right ventral cirri contribute to the formation of the FVTA VI, which forms the new right ventral cirral row for the daughter cells. Within these taxa, a short RVR is present in all *Hemiamphisiella* species but absent in all *Strongylidium* species and most *Pseudouroleptus* species (except *P. plestiensis*). We speculate that the length of the RVR may be phylogenetically informative for resolving evolutionary relationships of taxa within the family Strongylidiidae.

Berger (2008) indicated that the absence of dorsal kinety fragmentation and caudal cirri at the end of dorsal kineties 1, 2, and 3 could be the plesiomorphies of the strongylidiids. We hypothesize a common ancestor of Strongylidiidae characterized by: FVTA VI developed de novo, FVTA VI involved in the formation of LVR; RVR generated by the posterior portion of FVTA VI; RVR long and commencing from the anterior end of the cell; fewer than four dorsal kineties (Table 5).

**Table 5:** Plesiomorphies and apomorphies considered in assessment of the phylogenetic relationships within the Strongylidiidae

	Plesiomorphies	Apomorphies
1	Without dorsal kinety fragmentation	With dorsal kinety fragmentation
2	FVTA VI developed de novo	FVTA VI developed intrakinetally
3	FVTA VI involved the formation of LVR	FVTA VI not involved the formation of LVR
4	RVR generated by posterior portion of FVTA VI	RVR generated by the whole FVTA VI
5	Fewer than four dorsal kineties	More than four dorsal kineties
6	RVR longer than half of cell length and commencing from the anterior end of cell	RVR shorter than half of cell length
7	Caudal cirri at the end of dorsal kineties 1, 2, 3	Caudal cirri at the end of dorsal kineties 1, 2
8	AZM gap absent	AZM gap present

*AZM* adoral zone of membranelles, *FVTA VI* frontal–ventral–transverse cirral anlage VI, *LVR* left ventral cirral row, *RVR* right ventral cirral row

### Supplementary Information

Below is the link to the electronic supplementary material.

Supplementary file1 (PDF 231 KB)

Supplementary file2 (DOCX 19 KB)

## Data Availability

Zoobank number for the present work: urn:lsid:zoobank.org:pub:084DA53A-12E1-44E4-8922-CC037D0E0B77. Zoobank number for the genus *Heterouroleptus* gen. nov.: urn:lsid:zoobank.org:act:F1BE78C9-3BB6-4DF2-AC90-944B74F32A06. ZooBank number for *Heterouroleptus weishanensis* gen. nov., sp. nov.: urn:lsid:zoobank.org:act:9C03872A-5E45-4DCD- 846E- EF2B386AFB89. The newly sequenced SSU rDNA, ITS1-5.8S rDNA-ITS2, LSU rDNA, and COI genes in this study were deposited in the GenBank database with accession numbers as follows: OR666141 (*H. weishanensis* gen. nov., sp. nov.) and OR666142 (*S. wuhanense*) for SSU rDNA; OR666143 (*H. weishanensis* gen. nov., sp. nov.) and OR666144 (*S. wuhanense*) for ITS1-5.8S rDNA-ITS2; OR666145 (*H. weishanensis* gen. nov., sp. nov.) and OR666146 (*S. wuhanense*) for LSU rDNA. OR678690, OR678691 (*H. weishanensis* gen. nov., sp. nov.) and OR678692, OR678693 (*S. wuhanense*) for the COI gene.
