# Supplementary material for: Multi-gene-based investigation on the molecular phylogeny of the hypotrichous family Strongylidiidae (Protista, Ciliophora), with notes on the ontogeny of a new genus and new species
Source: Mar Life Sci Technol. 2024 Aug 23;6(3):442–61. doi: 10.1007/s42995-024-00243-z (PMC11358561; doi:10.1007/s42995-024-00243-z)
Supplement: Supplementary file 1 — Supplementary file1 (PDF 231 KB) [file 42995_2024_243_MOESM1_ESM.pdf]

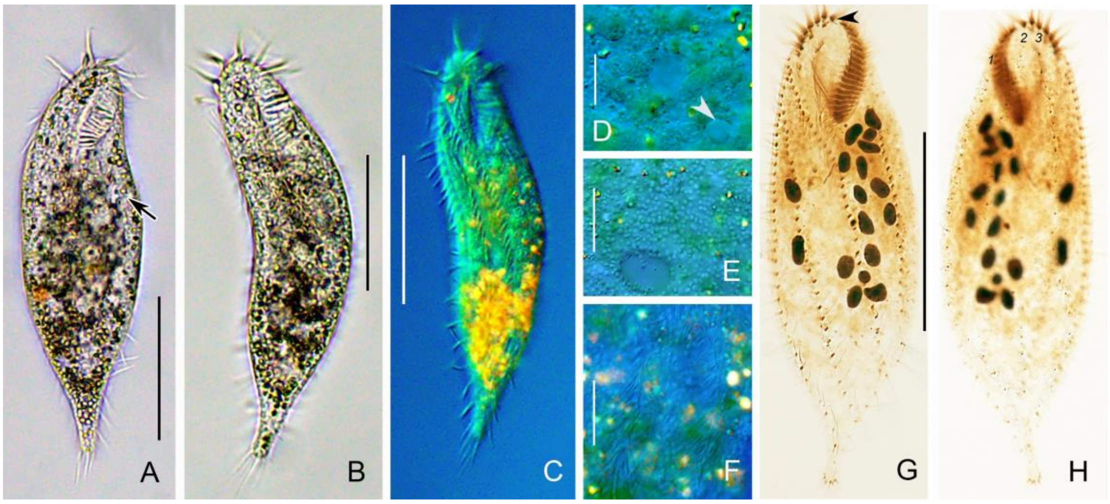

Supplementary Fig. S1 Photomicrographs of *Strongylidium wuhanense* from life (**A–F**) and after protargol impregnation (**G**, **H**). (**A**, **B**), bright field; (**C–F**), differential interference contrast. **A–C** Ventral view of different individuals, arrow in (**A**) indicates contractile vacuole. **D** Showing the micronuclei (arrowhead). **E**, **F** Showing the arrangement of cortical granules on the dorsal (**E**) and ventral (**F**) side. **G**, **H** Ventral (**G**) and dorsal (**H**) view of a representative specimen, to show the infraciliature and nuclear apparatus, arrowhead in (**G**) demonstrates the gap between two parts of adoral zone of membranelles. 1–3, dorsal kineties 1–3. Bars: 10 μm (**D**), 20 μm (**E**, **F**), 50 μm (**A–C**, **G**, **H**).
