# Supplementary material for: Multi-gene-based investigation on the molecular phylogeny of the hypotrichous family Strongylidiidae (Protista, Ciliophora), with notes on the ontogeny of a new genus and new species
Source: Mar Life Sci Technol. 2024 Aug 23;6(3):442–61. doi: 10.1007/s42995-024-00243-z (PMC11358561; doi:10.1007/s42995-024-00243-z)
Supplement: Supplementary file 2 — Supplementary file2 (DOCX 19 KB) [file 42995_2024_243_MOESM2_ESM.docx]

**Table S1** Morphometric data of *Strongylidium wuhanense* (Weishan population)

| Character | Min | Max | Mean | M | SD | CV | n |
| --- | --- | --- | --- | --- | --- | --- | --- |
| Length of cell in μm | 116 | 176 | 139.9 | 135 | 16.7 | 11.9 | 21 |
| Width of cell in μm | 29 | 54 | 41.5 | 40 | 7.3 | 17.7 | 21 |
| Cell width: length, ratio (percentage) | 22.0 | 41.7 | 29.7 | 30.2 | 4.4 | 14.9 | 21 |
| Length of AZM in μm | 31 | 41 | 34.1 | 34 | 2.9 | 8.6 | 21 |
| AZM: cell length, ratio (percentage) | 22.0 | 29.7 | 24.5 | 23.7 | 2.1 | 8.4 | 21 |
| Adoral membranelles 1, number | 6 | 7 | 6.2 | 6 | 0.4 | 6.5 | 21 |
| Adoral membranelles 2, number | 17 | 21 | 18.8 | 19 | 1.1 | 6.0 | 21 |
| Frontal cirri, number | 3 | 4 | 3.0 | 3 | 0.2 | 7.2 | 21 |
| Buccal cirrus, number | 1 | 1 | 1.0 | 1 | 0 | 0 | 21 |
| Parabuccal cirrus, number | 1 | 1 | 1.0 | 1 | 0 | 0 | 21 |
| Post-peristomial ventral cirrus, number | 1 | 1 | 1.0 | 1 | 0 | 0 | 21 |
| Left ventral cirri, number | 27 | 37 | 33.4 | 34 | 2.8 | 8.5 | 21 |
| Right ventral cirri, number | 19 | 37 | 26.4 | 27 | 4.2 | 16.0 | 21 |
| Left marginal cirri, number | 24 | 38 | 29.1 | 29 | 3.4 | 11.5 | 21 |
| Right marginal cirri, number | 27 | 39 | 33.1 | 32 | 3.5 | 10.6 | 21 |
| Dorsal kineties, number | 3 | 3 | 3.0 | 3 | 0 | 0 | 21 |
| Dikinetids in DK1, number | 18 | 29 | 23.0 | 23 | 2.9 | 12.7 | 21 |
| Dikinetids in DK2, number | 20 | 30 | 24.1 | 24 | 2.6 | 10.7 | 21 |
| Dikinetids in DK3, number | 20 | 26 | 23.6 | 24 | 2.1 | 8.9 | 21 |
| Macronuclear nodules, number | 15 | 22 | 16.7 | 16 | 2.0 | 11.7 | 21 |
| Length of anterior macronuclear nodule in μm | 5 | 11 | 7.6 | 8 | 1.7 | 22.0 | 21 |
| Width of anterior macronuclear nodule in μm | 4 | 7 | 4.7 | 5 | 0.9 | 18.6 | 21 |
| Length of posterior macronuclear nodule in μm | 5 | 11 | 7.6 | 7 | 1.8 | 23.8 | 21 |
| Width of posterior macronuclear nodule in μm | 4 | 6 | 4.9 | 5 | 0.6 | 12.9 | 21 |
| Micronuclei, number | 2 | 4 | 2.4 | 2 | 0.6 | 24.8 | 21 |
| Length of anterior micronuclei | 4 | 5 | 4.6 | 5 | 0.3 | 7.5 | 21 |
| Length of posterior micronuclei | 4 | 5 | 4.5 | 5 | 0.3 | 6.8 | 21 |

All data are based on protargol-impregnated specimens. Abbreviations: AZM, adoral zone of membranelles; CV, coefficient of variation in %; DK1–3, dorsal kineties 1–3; M, Median; Max, maximum; Mean, arithmetic mean; Min, minimum; n, number of cells measured; SD, standard deviation.
